# Spatial architecture of the immune microenvironment orchestrates tumor immunity and therapeutic response

**DOI:** 10.1186/s13045-021-01103-4

**Published:** 2021-06-25

**Authors:** Tong Fu, Lei-Jie Dai, Song-Yang Wu, Yi Xiao, Ding Ma, Yi-Zhou Jiang, Zhi-Ming Shao

**Affiliations:** 1 Department of Breast Surgery, Fudan University Shanghai Cancer Center, Shanghai, 200032 China; 2 Key Laboratory of Breast Cancer in Shanghai, Department of Oncology, Shanghai Medical College, Fudan University, Shanghai, 200032 China

**Keywords:** Tumor immunity, Tumor immune microenvironment, Spatial architecture, Immunotherapy

## Abstract

Tumors are not only aggregates of malignant cells but also well-organized complex ecosystems. The immunological components within tumors, termed the tumor immune microenvironment (TIME), have long been shown to be strongly related to tumor development, recurrence and metastasis. However, conventional studies that underestimate the potential value of the spatial architecture of the TIME are unable to completely elucidate its complexity. As innovative high-flux and high-dimensional technologies emerge, researchers can more feasibly and accurately detect and depict the spatial architecture of the TIME. These findings have improved our understanding of the complexity and role of the TIME in tumor biology. In this review, we first epitomized some representative emerging technologies in the study of the spatial architecture of the TIME and categorized the description methods used to characterize these structures. Then, we determined the functions of the spatial architecture of the TIME in tumor biology and the effects of the gradient of extracellular nonspecific chemicals (ENSCs) on the TIME. We also discussed the potential clinical value of our understanding of the spatial architectures of the TIME, as well as current limitations and future prospects in this novel field. This review will bring spatial architectures of the TIME, an emerging dimension of tumor ecosystem research, to the attention of more researchers and promote its application in tumor research and clinical practice.

## Background

Over the past few centuries, the concept of tumor has evolved from a simple aggregation of abnormally proliferating cells into a highly organized “organ”. Various components that compose tumors are termed the tumor microenvironment (TME) (1). Although the specific composition of the TME varies between tumor types, most of them share hallmark characteristics, including tumor cells, immune cells, stromal cells, extracellular matrix (ECM), vessels, soluble factors, and physical properties (Table 1) (2–5). Within the TME, all immune components are specifically defined as the tumor immune microenvironment (TIME) because of their unique internal interactions and essential roles in tumor biology, which comprises innate immune cells, adaptive immune cells, extracellular immune factors, and cell surface molecules (4, 6, 7). Studies have focused on the composition of immune cells in the TIME, and established mature theories and clinical applications (4, 8). For example, triple-negative breast cancer (TNBC) with more T cell infiltration generally presents better prognosis than those with less T cell inflamed (9). However, findings that patients with similar compositions of infiltrating immune cells have different prognoses are not well explained (10, 11), suggesting further exploration is needed on the TIME.

**Table 1:** Tumor microenvironment components

Components	Classification	Molecular characteristics		Function in TME	Major Role
*Immune cells*					
T lymphocytes					
CD8^+^ T cell	TME & TIME	CD3^+^CD8^+^		Cytotoxicity	Anti-tumoral
Th1 cell	TME & TIME	CCR6^+^CXCR3^+^	Production of IL-2 and IFN-γ	CD8^+^ T cell supporting	Anti-tumoral
Th2 cell	TME & TIME		Production of IL-4, IL-5 and IL-13	B cell responses supporting	Pro-tumoral
Th17 cell	TME & TIME		Production of IL-17A, IL-17F, IL-21, IL-22	Angiogenesis, tumorigenesis, immune regulation	Ambiguous
Treg cell	TME & TIME	CD4^+^FOXP3^+^CD25^+^		Immune suppression	Pro-tumoral
B lymphocytes	TME & TIME	CD19^+^CD20^+^CD138^+^		Antibody production, antigen presentation, TLSs formation	Anti-tumoral
Innate lymphoid cell (ILCs)					
ILC1s					
NK cells	TME & TIME	Expression of T-bet; Eomes^+^ CD27^−^		Cytotoxic functions without prior sensitization	Anti-tumoral
Non-NK cells	TME & TIME	Expression of T-bet; Eomes^−^ CD27^+^		Secretion of IFN-γ and TNF-α to induce tumor dependent anti-tumor immune responses	Ambiguous
ILC2s	TME & TIME	Expression of GATA3; secretion of type 2 cytokines		Secretion of IL-5, IL-13, IL-4 to inhibit anti-tumor immunity	Pro-tumoral
ILC3s	TME & TIME	Expression of RORγt		Heterogeneous cells secreting various cytokines and chemokines	Ambiguous
Tumor-associated macrophages		HLA-DR^+^CD68^+^ CD11c^−^			
M1	TME & TIME	CD86^+^ CD80^+^ iNOS^+^		Promoting anti-tumor T_H_1 and T_H_17 immune responses	Anti-tumoral
M2	TME & TIME	CD163^+^CD206^+^		Supporting angiogenesis, tumor progression, and metastasis; immune suppression	Pro-tumoral
MDSCs	TME & TIME	CD11b^+^HLA-DR^−^		Differentiating into TAMs, immune suppression	Pro-tumoral
Dendritic cells	TME & TIME	Siglec-H^+^CD317^+^		Binary immune regulatory function shaped by TME	Ambiguous
Neutrophils		CD14^+^HLA-DR^+^CD206^−^CD86^−^			
N1	TME & TIME	Supported by TGF-β and G-CSF		Immune respond initiation, antigen presenting	Anti-tumoral
N2	TME & TIME	Supported by IFN-γ and hepatocyte growth factor		Remodeling ECM, promoting angiogenesis and tumor growth	Pro-tumoral
*Stromal cells*					
Cancer-associated fibroblasts	TME	Cellular markers: α-SMA, FAP-α, FSP-1/S100A4, PDGFRβ		Promoting tumor cell proliferation and invasion, angiogenesis; ECM remodeling; bidirectional immune regulation	Pro-tumoral
Endothelia cells	TME	Cellular markers: CD31; consisting of blood vessels		Angiogenesis, tumor metastasis	Pro-tumoral
Pericytes	TME	Cellular markers: Calponin, CD90, DLK, NG2, PDGF-A, SMA		Promote primary tumor growth, negative regulator of metastasis,	Pro-tumoral
Adipocytes	TME	Cellular markers: CD34		Secretion of hormones, metabolites, growth factors, enzymes and cytokines	Pro-tumoral
Mesenchymal stem cells	TME	Cellular markers: CD105, CD90, CD117, CD133		Forming the premetastatic niche; promoting tumor initiation and progression	Pro-tumoral
*Vessels*					
Blood vessel	TME	Tubular structures formed by endothelial cells (cellular markers: CD31)		Promoting metastasis and angiogenesis,	Pro-tumoral
Lymph vessel	TME	Tubular structures formed by lymphatic endothelial cells (cellular markers: LYVE-1, Podoplanin, PROX1, VEGFR-3)		Foster tumor metastasis, physical link between lymph nodes and tumor	Pro-tumoral
*Extracellular matrix*					
Collagen, fibronectin, elastin, laminin	TME	Complex noncellular three-dimensional macromolecular network		Physical scaffold for cell, tumor cell dissemination, depot for cytokines and growth factors	Pro-tumoral
Matrix metalloproteases, Cathepsins	TME	Enzymes secreted and activated by malignant cells in extracellular matrix		ECM remodeling, aiding metastasis, angiogenesis and inflammation	Pro-tumoral
*Immune molecules*					
Cytokines	TME & TIME	Mainly secreted by immune cells; acting in a paracrine, autocrine or endocrine manner		Promoting leukocyte growth, survival and activation; promoting tumor immunogenicity, tumor cell proliferation, angiogenesis	Ambiguous, mainly anti-tumoral
Chemokines	TME & TIME	Regulation of cell movement and leukocyte attractants		Promoting tumor cell migration, invasion and metastasis; promoting immune cell migration, maturation	Ambiguous, mainly anti-tumoral
*Extracellular nonspecific chemicals*					
Oxygen	TME	Aerobic cellular respiration		Favor immunosuppressive phenotypes	Ambiguous
Amino acids	TME	Components and substrates for various critical processes in cell metabolism and physiology		Supporting physiological processes of both tumor and immune cells, immune regulation	Ambiguous
Glucose	TME	Major source of energy for cells		TME dependent influence of energy metabolism in both immune and tumor cells	Ambiguous
Lactate	TME	Products of glycolysis		Inhibiting anti-tumor immunity, decrease the pH within the TME	Pro-tumoral
Carbon dioxide	TME	Products of oxidative phosphorylation		Causing tumor tissue acidosis	Ambiguous
Fatty acids	TME	Involvement in biological progression and cell structure		Heterogeneous consequences for different cells, leading to immunosuppressive effects	Ambiguous
Metal ions	TME	Involvement in biological progression		Regulating tumor and immune cells	Ambiguous

TME, tumor microenvironment; TIME, tumor immune microenvironment; MDSCs, myeloid-derived suppressor cells; TLSs, tertiary lymphoid structures

**Fig. 1:**
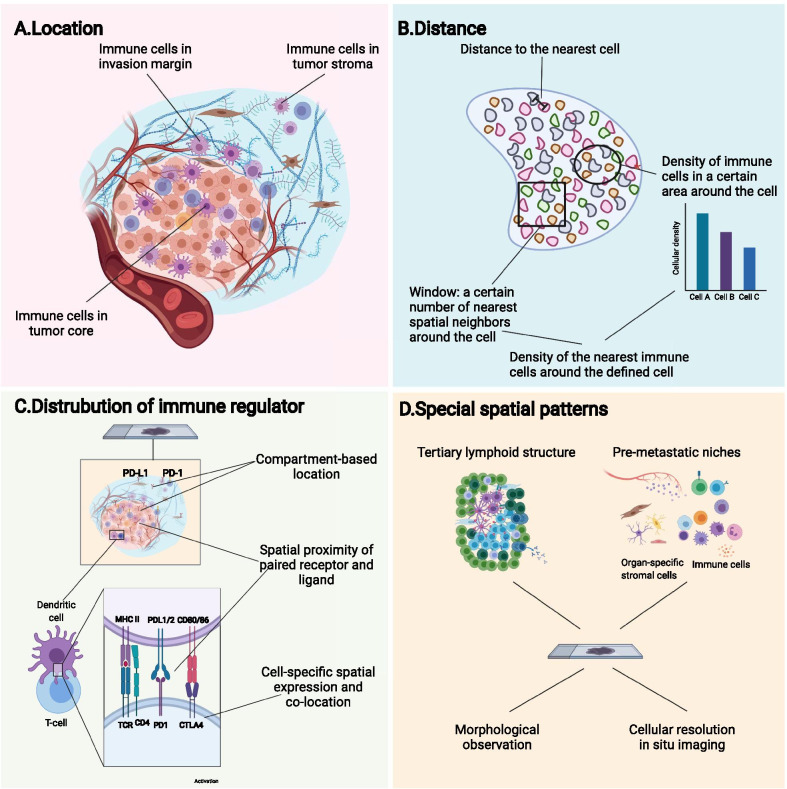
Definition and components of the spatial architecture of the tumor immune microenvironment (TIME). The spatial architecture is described according to the location of immune cells (**a**), distance between cells (**b**), distribution of immunoregulators (**c**), and specific spatial patterns (**d**)

**Fig. 2:**
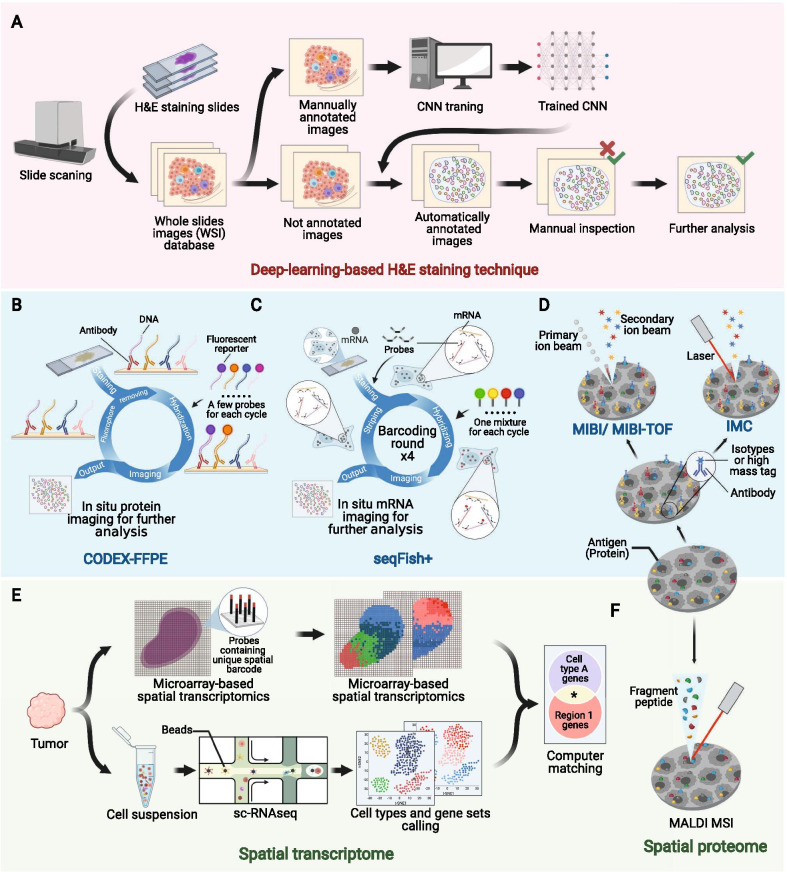
Emerging techniques used to identify the spatial architecture of the tumor immune microenvironment. Pink area (**a**), deep-leaning-based HE techniques. Blue area (**b**–**d**), probe-based in situ technologies. **b**, CODEX-FFPE; **c**, seqFISH+ ; **d**, IMC and MIBI/MIBI-TOF. Green area (**e**–**f**) spatial omics. **e**, microarray-based spatial transcriptomics + sc-RNA-seq; **f**, MALDI MSI. Consult Table 3 for more detailed information. H&E, hematoxylin and eosin; CNN, convolutional neural network; MALDI MSI, matrix-assisted laser desorption/ionization mass spectrometric imaging; UV, ultraviolet; sc-RNAseq, single-cell RNA sequencing; IMC, imaging mass cytometry; MIBI, multiplexed ion beam imaging; MIBI-TOF, multiplexed ion beam imaging by time of flight; CODEX, codetection by indexing; FFPE, formalin-fixed and paraffin-embedded

**Fig. 3:**
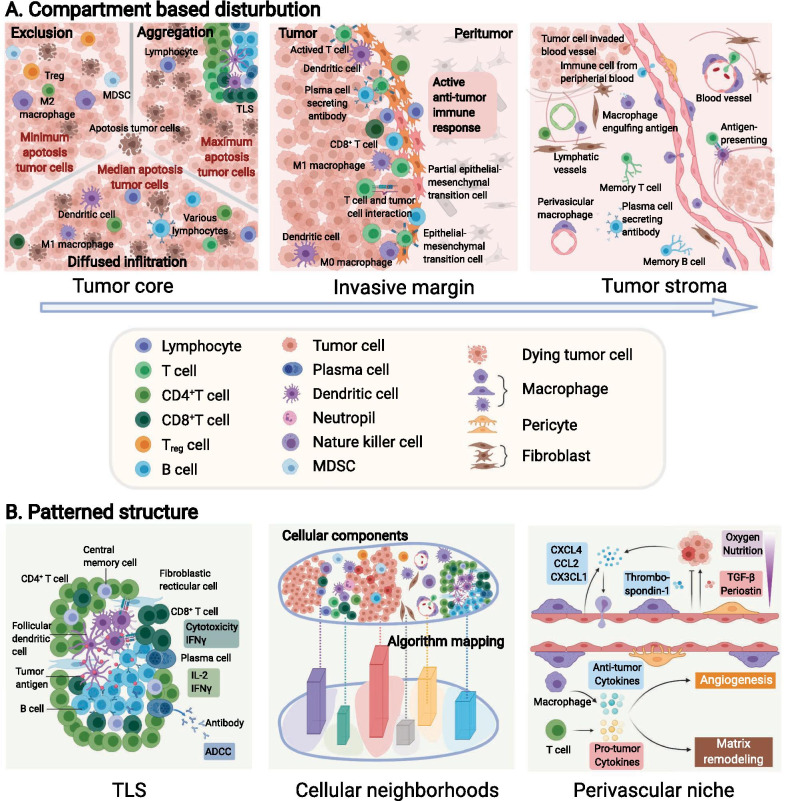
Representative spatial architecture of immune cells in the tumor microenvironment. **a** Primary tumors are divided into the tumor core, tumor stroma, and invasion margin based on tumor compartments. **b** Special immune structures, such as perivascular niches and tertiary lymphoid structures (TLSs), are also involved in the construction of architectures. Moreover, computational technology identified cellular neighborhoods (CNs) as regions with a characteristic local stoichiometry of cellular components. CXCL4, C-X-C chemokine ligand type 4; CCL2, C–C chemokine ligand type 2; CX3CL1, C-X3-C motif ligand 1; TGF-β, transforming growth factor-β; IFN, interferon; IL-2, interleukin 2; ADCC, antibody-dependent cellular cytotoxicity; MDSC, myeloid-derived suppressor cell

**Fig. 4:**
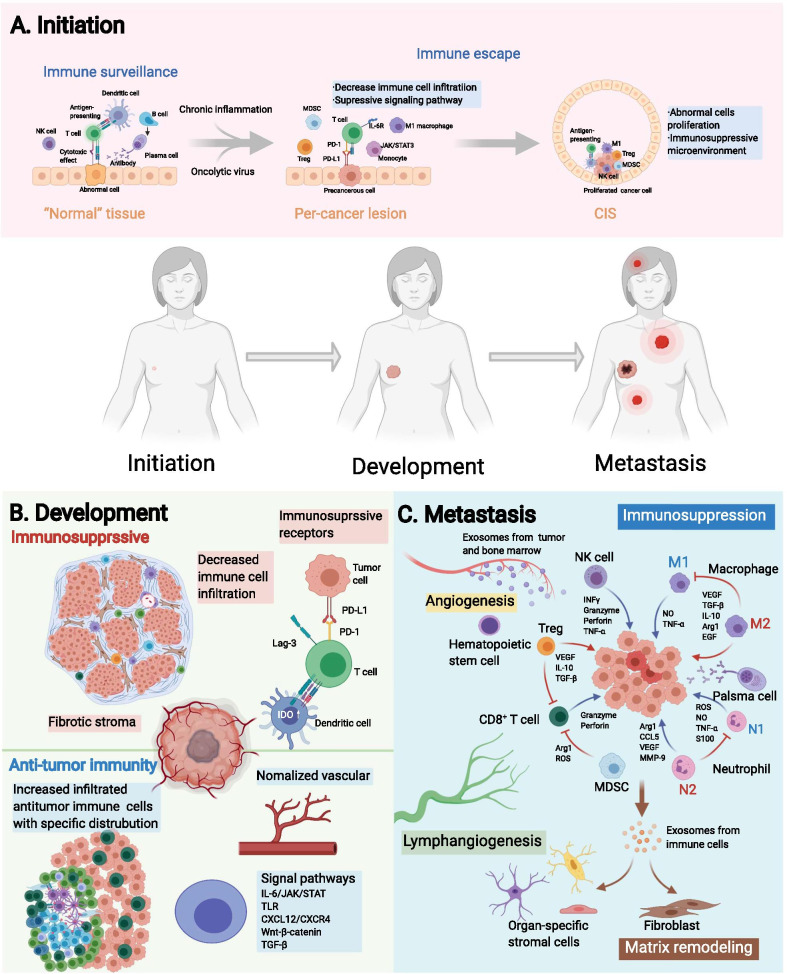
Spatial evolution of the tumor immune microenvironment (TIME) structure during tumor progression. The process of tumor initiation, expansion, and metastases is accompanied by a gamble between the tumor and the TIME, where antitumor immune and immunosuppressive factors coexist and interact with each other. **a** In the initiation stage, the immune components around the lesion evolve from immune surveillance to immune escape during the evolution of "normal tissue", precancerous lesions, and carcinoma in situ (CIS). **b** In the expansion phase, the TIME functions in a contact-dependent or distance-dependent manner. **c** In the metastatic phase, the specific arrangement of immune cells in the metastatic niche establishes a favorable environment for the formation and growth of metastases. PD-1, programmed cell death 1; PD-L1, programmed cell death ligand 1; Lag-3, lymphocyte-activation gene 3; TLSs, tertiary lymphoid structures; TLR, Toll-like receptor; CXCL12, C-X-C chemokine ligand type 12; CXCR4, C-X-C chemokine receptor type 4; TGF-β, transforming growth factor-β; IL, interleukin; VEGF, vascular endothelial growth factor; ROS, reactive oxygen species; NO, nitric oxide; EGF, endothelial growth factor; Arg1, Arginase-1; CCL5, C–C chemokine ligand type 5; TNF-α, tumor necrosis factor α

To obtain diversified information about the TIME, emerging researches focus on not only its compositions and molecular features, but also the spatial organization of components in the TIME, summarized as its spatial architecture. The spatial architecture of the TIME is now described mostly from the following four aspects (Fig. 1 and Table 2): (1) location of immune cells in the tumor compartments (Fig. 1a); (2) distance between cells, evaluated by the distance between cell and its nearest neighborhood (10), or the density of cells around the defined cell (12) (Fig. 1b); (3) spatial distribution of immune regulators (Fig. 1c); (4) spatial patterns formed by the well-organized TIME components characterized morphologically and molecularly (Fig. 1d). Moreover, distance-dependent immune interactions like paracrine, autocrine or cell contact also support that spatial relation matters in the research of TIME. While some previous studies have provided insights into the spatial architecture of the TIME using conventional techniques (13, 14), innovative technologies have portrayed the TIME with higher flux, higher dimensionality and higher resolution ratio. The evolved knowledge about the spatial architecture facilitated revealing its effect on the clinical prognosis and immunotherapy efficacy (10, 15–21).

**Table 2:** Description and characterization of the spatial architecture of the tumor immune microenvironment (TIME)

Components	Definition	Detection methods	Detecting characteristics
Location of immune cells	Identification and quantification of immune cells in different compartments of tumor	H&E staining, digital pathology	Morphological differences; visual distinction of tumor compartments (i.e., sTILs, iTILs)
		Probe-based in situ imaging	Cellular markers
		Spatial omics	Expression signature
Distance between immune cells	The shortest distance between cells	Cellular-resolution imaging and analysis algorithms	The recognition and identification of cells and their surrounding cells; determination of distance between cells
	Density of immune cells in a certain area around the tumor cell		
Distribution of immune regulators	Compartment-based distribution	Probe-based in situ imaging and/or spatial omics	Spatial protein or mRNA expression at cellular or subcellular resolution
	Cell-specific spatial expression and co-location		
	Spatial proximity of paired receptor and ligand		
Identification of specific spatial patterns	Robust spatial architecture of immune cells with specific aggregation and distribution patterns (i.e., TLSs, peri-vascular niches)	Digital pathology	Visual spatial arrangement features of cells
		Immunohistochemistry	Pattern-specific marker
		Probe-based in situ imaging and/or spatial omics	Pattern-specific marker at cellular or subcellular resolution

TILs, tumor infiltrated lymphocytes; TLSs, tertiary lymphoid structures

In this review, we will first summarize the emerging spatial analysis technologies, together with their features and scope of application. Methods used by scientists to describe the complex architectural traits of the TIME are subsequently discussed. Next, we discuss the implication of the TIME architectures in tumor initiation, expansion, invasion and metastasis. We also included gradients of extracellular nonspecific chemicals (ENSCs), a regulatory factor usually ignored in the TIME, to emphasize its importance in TIME function. Then, we reviewed the clinical potential of the spatial architecture of the TIME. Finally, discussions and recommendations for methods to overcome current setbacks and future development in this field are proposed.

## Emerging technologies used to characterize the spatial architecture of the TIME

Emerging technologies with a higher flux, higher dimensionality and higher resolution ratio have extensively broadened researchers’ horizon of the spatial architecture of the TIME, which has not been fully characterized previously due to its microcosm and complexity. Here, we reviewed some representative emerging technologies for identifying the spatial architecture of the TIME (Fig. 2 and Table 3) (10, 19, 22–41), which might facilitate further mechanistic research.

**Table 3:** Emerging techniques to identify spatial structure of the tumor immune microenvironment (TIME)

Category	Name	Level	Sample preparation	Labels	Visualization	Comments	Refs.
Non-specific technique	H&E staining	Nonspecific structure	FFPE	Hematoxylin and eosin	Visible light	Low-cost; high flux helped by deep learning: poordiscrimination of cell subtypes	[22–25]
Probe-based in situ imaging	IHC	Peptide and protein	FFPE	Antibody-reporter (usually florescent protein)	Fluorescence, insoluble pigment, etc.	Limited detectable targets simultaneously	[26]
	FISH	DNA	FFPE	Oligonucleotides-florescent reporter	Fluorescence	Limited detectable targets simultaneously	[27]
	CODEX	Peptide and protein	Fresh-frozen	Antibody-oligonucleotides	Fluorescence	Extended detectible targets simultaneously	[28]
	CODEX-FFPE	Peptide and protein	FFPE	Antibody-oligonucleotides	Fluorescence	Extended detectible targets simultaneously	[10]
	seqFISH, seqFISH + , corFISH	mRNA (sub-transcriptome)	Live section	Oligonucleotides-florescent reporter	Fluorescence	> 10,000 detectible genes simultaneously; subcellular resolution ratio	[29–31]
	MIBI, MIBI-TOF	Peptide and protein	FFPE, immobilized cell suspension	Antibodies-isotypes	Secondary ion beam	Extended detectible targets simultaneously	[19, 32, 33]
	IMC	Protein, etc.	FFPE	Antibodies-high mass tag	Ion beam	Subcellular resolution ratio; probes are not necessarily needed	[34, 35]
Spatial transcriptome	Microarray-based spatial transcriptomics	Transcriptome	Live section, FFPE	Spatial transcriptome	Pathology + Computational analysis	Restricted cellular resolution	[36]
	Microarray-based spatial transcriptomics + sc-RNAseq	Transcriptome	Live section + single-cell suspension	Spatial transcriptome	Computational matching	Mapping is based on region-cluster matching by multimodal intersection analysis	[37]
	ZipSeq	Transcriptome	Live section	Antibody/lignoceric-oligonucleotides-zipcode	Computational matching	Exquisite design in advance is required for accuracy	[38]
Spatial proteome	MALDI MSI	Proteome	Fresh frozen, FFPE	–	Ion imaging	Label-free; de novo investigation	[39, 40]

H&E, hematoxylin and eosin; FFPE, formalin-fixed and paraffin-embedded; IHC, immunohistochemistry; FISH, fluorescence in situ hybridization; DNA, deoxyribonucleic acid; CODEX, co-detection by indexing; MIBI, multiplexed ion beam imaging; MIBI-TOF, multiplexed ion beam imaging by time of flight; IMC, imaging mass cytometry; sc-RNAseq, single-cell RNA sequencing; MALDI MSI, matrix-assisted laser desorption/ionization mass spectrometric imaging

### Deep learning methods for H&E staining slides

Hematoxylin and eosin (H&E) staining, which is one of the most common techniques adopted by pathologists, has now been rekindled by deep learning method. Conventionally, pathologists observe the spatial architecture of the TIME in formalin-fixed and paraffin-embedded (FFPE) or fresh-frozen samples under a microscope (42–44). However, manual observation of slices is laborious and may result in considerable interobserver discrepancies (45, 46). The introduction of deep learning methods based on whole slide images (WSIs) can automate and standardize the process.

The convolutional neural network (CNN), a universal methodology for processing medical images (47–50), is the most commonly used algorithm for processing WSIs (Fig. 2a). Basically, before adopting CNN, the program must be trained with manually annotated images according to recognition purposes, e.g., marking out certain types of cells or regions. Then, the trained CNN can process coarse images and generate automatically annotated images. Next, a manual quality inspection step often follows to verify the results. Using the collected data, deeper investigations can be performed, such as cell quantification, spatial clustering, intercellular interaction analyses, significance testing with clinical phenotypes, and correlation predictions with sequencing data (22–25).

The integration of deep learning and routine H&E staining techniques can reveal surprising pathological traits that might have been previously ignored by human eyes, with nearly no extra cost and impressive accuracy and efficacy. Moreover, the high-flux deep-learning-based analysis might revive prodigious historical databases of H&E-stained samples, which is promising for large-scale retrospective studies. However, despite the convenience and low cost of H&E staining, it is usually not capable of classifying specific subtypes of immune cells in the TIME.

### *Probe-based *in situ* imaging*

Discriminating immune cell subtypes by biomarker-probe pairs has been well established, as represented by immunohistochemistry (IHC) and fluorescence in situ hybridization (FISH) (51, 52). One primary restriction of IHC or FISH is the limited quantity of detectable targets in one section because of the overlap of the emission spectrum of fluorescent reporters. In addition, the techniques arouse the concern of consuming too many precious sections if the detection of a large number of targets is required. To solve these problems, two technology roadmaps were conceived: one by batching the imaging of fluorescent signals and the other by substituting electromagnetic wave with particle flow.

For the first strategy, several methods were proposed. Adopted from Codetection by indexing (CODEX) (28), CODEX-FFPE is a system based on special antibodies conjugated with single-strand DNA, which is suitable for both FFPE samples and tissue microarrays (Fig. 2b). The target protein is detected by DNA-conjugated antibodies, and then, imaging cycle starts. In each imaging cycle, only a few kinds of complementary DNA chains linked with different fluorescent reporters are added to image. Then, these complementary DNA chains are striped to undergo another cycle. And there have been commercial version of CODEX-FFPE provided (10). CorFISH (29), seqFISH + (30) and seqFISH (31) are a series of technologies that exploit two classes of oligonucleotide DNA probes (Fig. 2c). Each primary probe contains 5 domains including one for binding target mRNA and 4 for binding secondary probes. In each cycle of imaging, an exquisitely predesigned mixture of secondary probes labeled with different fluorescence reporters is added, and thus, mRNAs will be marked with different colors. Then, these secondary probes are eluted, and a new hybridization cycle starts. The mRNAs will be hybridized with another mixture of secondary probes. After several cycles, nucleotide sequences of mRNA molecules in situ can be deciphered from the unique array of colors in corresponding location.

Mass spectrometry is another strategy for improving IHC and FISH (53, 54). While signal of fluorescent reporters consists of a consecutive range of light spectrum, mass spectrometric signal is discrete peaks, endowing it with higher resolution to discriminate different reporters. Besides, the cell-by-cell scanning manner used in mass-spectrometry-based techniques can also achieve a higher spatial resolution. Multiplexed ion beam imaging (MIBI) (19) and multiplexed ion beam imaging by time of flight (MIBI-TOF) (32, 33) take advantage of antibodies labeled with isotypes (Fig. 2d). And during scanning, these isotypes can be excited by primary oxygen ion beam to form secondary ion beam. Another technique called imaging mass cytometry (IMC) works in an analogous way but uses laser to activate isotypes (Fig. 2d). And notably, IMC does not necessarily need antibody probes to label cells, but also uses endogenous or exogenous tissue-specific chemicals, such as iodine isotopes in the thyroid, to achieve imaging (34, 35).

After the generation of raw images, pathologists can perform further processing and refinement, such as the segregation of cells, classification of immune cells and other analyses, facilitated by bioinformatics tools. Probe-based in situ technology is characterized by its detailed preservation of spatial information and relatively low cost in large-scale applications. Nevertheless, probe-based technologies only detect known targets with existing probes. Thus, they are not suitable for detecting novel biological events, such as undefined molecules.

### Spatial transcriptome and proteome

Omics techniques are ideal for conducting de novo investigations or revealing the landscape of the TIME due to their probe-free traits, high flux, and large capacity. Currently, the spatial transcriptome and spatial proteome are the two most commonly used spatial omics technologies.

One representative kind of spatial transcriptome is microarray-based spatial transcriptomics (Fig. 2e) (36). On the microarray chip used for the detection of histological sections, there are massive probes containing unique positional barcodes for locating. The barcodes can be sequenced in the subsequent sequencing, thus enabling the mapping of transcriptome information to histological sections. Microarray-based spatial transcriptomics combines transcriptome and histology to extend transcriptome to a 2-D dimension, and has been commercialized and adopted in the researches of several malignancies (55–58). However, the spatial resolution of microarray-based spatial transcriptomics is limited that it only has a minimal resolution power of discriminating 10–200 cells. Thus, Moncada et al. (37) combined microarray-based spatial transcriptomics with single-cell RNA sequencing (scRNA-seq) to integrate their advantages. With reference maps generated from microarray-based spatial transcriptomic, data from the scRNA-seq of the rest of tissue can be mapped back.

There are also some other technology roadmaps in spatial transcriptomics, such as ZipSeq. Researchers first hybridized live tissue with cell-marker-specific antibody probes containing unique zipcode oligonucleotides that are initially blocked by photocleavable protecting group. Then, region of interest (ROI) of any shape was radiated with ultraviolet to remove blocking groups, making it possible to combine with complementary oligonucleotides conjugated to fluorescent reporters. Then, processed tissue was imaged to obtain reference map and digested to undergo scRNA-seq, whose outcome will be matched back to the reference map ultimately (38).

Apart from the spatial transcriptome, the spatial proteome represented by matrix-assisted laser desorption/ionization mass spectrometric imaging (MALDI MSI) is also capable of depicting in situ omics information (Fig. 2f). As a label-free system, although the spatial proteome also takes advantage of mass spectrometry for imaging, the excitation beam in the spatial proteome is much stronger and is able to directly ionize the components of sections and preserve spatial information simultaneously during scanning (39, 40).

Spatial omics has enabled researchers to better understand the actual biological events occurring in the architecture of the TIME. Spatial omics not only quantifies and locates immune cells but also further reveals their functional status and potential intercellular reactions. However, concern persists about the matching accuracy and precision of spatial omics. In addition, complex protocols and sizable expenses are also setbacks and challenges for large-scale studies or clinical use.

### Software for TIME analysis

In-depth data mining is necessary to take full advantage of massive data derived from the techniques reviewed above. Some basic functions, such as cell segregation, classification, quantification and other primary extended tools, are inlayed to the solutions mentioned above. In addition, developers have also proposed general analysis software for deeper analyses. For instance, spatial variance component analysis is a computational algorithm able to analyze cell–cell communication in spatial architecture of the TIME. It deconstructs overall effects that cells receive into four aspects: intrinsic effects, environmental effects, cell–cell interactions, and residual noise (17). In the near feature, an explosion of data is foreseeable as researchers increasingly focus on the spatial architecture of the TIME. More useful tools will be necessary for an in-depth analysis of these precious and complex data.

## Aspects to describe the spatial architecture of the TIME

Several strategies are proposed to profile complex TIME information generated by the innovative tools reviewed above or other conventional techniques (Table 2). Different strategies can provide us with different silhouettes to comprehend the complexity of the TIME.

### Distribution of immune cells based on tumor compartments

The most widely utilized mode to describe the distribution of immune cells is categorizing them according to the compartments in which they are located in the tumor tissue (Figs. 1a and  3a). The compartment in which immune cells reside potentially reflects their relationships with tumor cells, other immune cells and other various components within the TME (59).

Typically, tumor tissue compartments consist of three parts: the tumor core (TC, also referred to as the tumor nest or tumor cluster), which accommodates the majority of tumor cells in some bordered area; the tumor stoma (TS), where abundant stromal components are situated around TC; and the invasive margin (IM), which represents the transition zone of the TC and TS. These spatial compartments can be observed in a variety of solid tumors such as breast cancer, colorectal cancer, melanoma and oral-tongue cancer (60–64). Thus, the location of immune cells can be described based on the specific compartment. Several papers have previously reported that the distribution of immune cells in these three regions is not significantly different (65, 66), while others found differences in the distribution and functional status of immune cells among these different locations (67, 68). For example, immune cells within TCs are universally considered to have the tightest interaction with tumor cells due to their close juxtaposition to tumor cells (69). The IM is thought to be the front line of the battle between the tumor and immune system, and unsurprisingly, the density and function of effective immune cells at this site are higher than those in either the TC or TS (70, 71). Immune cells in the TS are involved in stromal remodeling and angiogenesis, which may exert profound effects on tumor growth and invasion (72).

Emerging imaging and analyses have further revealed intrinsic differences among specific types of immune cells within these three regions. For instance, TNBC is a highly aggressive and heterogeneous subtype of breast cancer. An analysis of spatial-based microdissection gene expression data in TNBC revealed the differences in tumor-infiltrating CD8^+^ T cells with respect to functional markers, interferon signatures, and immune inhibitory molecule cells among these three regions (59). Furthermore, T cells located in different compartments possess a heterogeneous T cell receptor repertoire (73) and exclusion gene expression (74, 75).

Although the specific definitions of the TC, IM and TS are slightly different from one study to another, describing the spatial architecture of TIME according to compartments has been widely acknowledged for its practicability, clarity and intuitiveness. And notably, there also exists heterogeneity in the spatial distribution of immune cells within these three regions, probably due to different tissue origin of tumor and high mobility of immune cells.

### Distance between immune cells and other cells in the TME

If we take all immune cells as a universal system (and this is exactly how they function in fact), describing immune cells according to the compartment they located in will actually carve up this kind of totality. Contrarily, measuring the distance between immune cells and other cells can provide a much more meticulous, precise and direct view to comprehend the spatial architecture of immune cells (Figs. 1b and 3a).

The distance between immune cells and tumor cells might directly reflect the lethality of immune cells toward tumors or, in contrast, the editing of immune cells by tumor cells (12, 76–79). The distance between different immune cells potentially reflects the ubiquitous interactions within immune cell populations and helps researchers to better understand all immune cells as a totality (80, 81). To date, the distance between immune cells and stromal cells is not well understood in this field. However, with more emphasis being attached to the stromal components of the TME, such as cancer-associated fibroblasts (CAFs) and tumor-associated adipocytes, a future trend might surge to investigate the effects of spatial relationships on the interactions between immune cells and stromal cells (82–84).

Nevertheless, the application of this parameter for describing the spatial architecture of immune cells also has its own deficiency, primarily because of its inconvenience and intricacy.

### Patterned structures of the TIME

When analyzing the distribution of immune cells inside tumors, some patterned structures composed of well-organized immune cells show relative intratumor, intertumor, and interpersonal consistency. These structures have been recognized in multiple tumors and were found to be of great clinical value (Figs. 1d and 3b).

Tertiary lymphoid structures (TLSs) are ectopic hyperplasia lymphoid tissues located outside the immune organs (85) that are present in a fraction of specific types of tumors. TLSs are composed of a T cell-rich zone containing mature dendritic cells (DCs), a CD20^+^ B cell-rich follicle with follicular DCs, plasma cells, and antibodies. Immune cells in TLSs activate antitumor immune response through antibody-dependent cellular cytotoxicity and/or a direct cytotoxic function. Recently, three studies were published back-to-back, further illustrating the localization, spatial composition and function of TLSs, as well as their relationship with the immunotherapy response and survival, highlighting the significance of TLSs as a new area worthy of further exploration (86–88).

Another group of special organization patterns of immune cells is a series of niches that are engaged in multiple biological processes, among which the perivascular niche is best described. The spatially heterogeneous blood flow within tumors constructs perivascular niches that supply oxygen, nutrients and growth factors, as well as remove toxic metabolites (89). According to previous studies, multiple behaviors of tumor cells and immune cells are regulated by regulatory factors in the perivascular niche (90–95). During tumor development, chemokines and cytokines in the TME attract circulating immune cells from the blood. Thus, as the first station of immune infiltration, the density of immune cells in the perivascular area is relatively high (96). Notably, macrophages might play an important role in the TIME around blood vessels due to their bilateral tumor-promoting or tumor-suppressive functions in multiple cancers (96–99). To our knowledge, many gaps remain in our understanding of other perivascular immune cells, which require further documentation.

Other niches, such as the stem cell niche, premetastatic niche and metastatic niche, are also proposed as novel concepts in tumor development, where immune cells seem to play an essential role. Related research is relatively sparse but has been growing rapidly over the past few years (100, 101), which is discussed in detail later in this review.

In addition to the aforementioned structures with certain morphological and pathological characteristics, the development of computational image processing has facilitated the identification of specific structures of the TIME that are undetectable with the human eye, which are termed cellular neighborhoods (CNs) (10). The algorithm-defined CNs helped to reveal the spatial organization of the TIME in different patients, and the results showed that the local aggregation of PD-1^+^ CD4^+^ T cells was associated with a better prognosis of high-risk patients. However, due to the black box of computational analysis, the recognizable biomarkers, biological characteristics, and functions of CNs in tumor immunity and therapeutic responses are unclear. Therefore, subsequent studies designed to reveal the biological nature and clinical application of CNs should be important.

### Spatial architecture of immune targets

In clinical application, usually it is more practical to detect certain immune targets than immune cells to obtain spatial information about the TIME. Thus, research in the spatial distribution of immune targets in the TIME has also attracted great attention (Fig. 1c).

Given the emerging prevalence of immunotherapies that modify antitumor immunity, a variety of immunomodulatory molecules, represented by immune checkpoints, are favorable immune targets (102). For example, antibodies targeting the T cell inhibitory checkpoint proteins programmed cell death 1 (PD-1), its ligand PD-L1, and cytotoxic T-lymphocyte-associated antigen 4 (CTLA-4) can enhance antitumor immunity. These antibodies have achieved durable clinical efficacy in some patients with various tumor types and have been approved by the U.S. Food and Drug Administration. The spatial localization of these immune targets has been measured in various cancers, including breast cancer (19, 59), non-small cell lung cancer (NSCLC) (103, 104), melanoma (105), colon cancer (106), and craniopharyngioma (107). Given the cell-specific expression of immune targets and well-organized spatial architecture of cells in the TME, the spatial distribution of these immune targets also showed a certain pattern (Fig. 1c). Taking the PD-1/PD-L1 axis as an example, PD-1 is mainly expressed on the surface of CD8^+^ T cells, while its ligand PD-L1 is expressed on multiple cells, including tumor cells, B lymphocytes, and tumor-associated macrophages (TAMs) (108–110). The results showed that the overall spatial distribution of immune targets is highly correlated with cells, although the levels of these targets vary among the same type of cells in different spatial locations (107).

Moreover, since most immunoregulators depend on ligand-receptor binding, the distance between two targets is crucial for the immune response. Studies have assessed the spatial interaction based on the distance between immune target positive cells, the spatial proximity of targets, or their colocalization in one cell (103, 105, 111). Additionally, taking PD-1/PD-L1 as an example, the PD-1/PD-L1 interaction score, an indicator of the spatial ligand-receptor relationship, was evaluated in patients treated with anti-PD-1. Patients with high interaction scores were more likely to respond, although individual biomarkers were not associated with the response or survival (110). Other tumor-related pathways, such as Notch, interleukin 6 (IL-6)/JAK/STAT, Toll-like receptor, C-X-C chemokine ligand type 12 (CXCL12)/C-X-C chemokine receptor type 4 (CXCR4), Wnt-β-catenin, and transforming growth factor (TGF-β), are also activated by a distance-dependent interaction between immune cells and their environment (112–116). Extracellular paracrine and juxtacrine signaling molecules also act in this manner (117). Furthermore, the relationship between spatial proximity and the functional statue of these signal pathways is awaited to be investigated.

## Spatial architecture of the TIME and tumor biology

The spatial architecture of the TIME is closely related to tumor biology, which coordinates with the development of tumors and simultaneously, exerts effects on tumors. Here, we reviewed the current knowledge of the spatial architecture and function of the TIME in tumor initiation, expansion and metastasis, to discuss the potential mechanism of the evolution of TIME’s spatial architecture and its implications for clinical outcomes (Fig. 4).

### Construction of the TIME at tumor initiation

The spatial distribution of immune components during tumor initiation has been described by some studies (Fig. 4a). Studies of the somatic mutation burden in morphologically normal precancerous tissues have revealed that “normal” cells accumulate a series of mutations prior to pathologically observable morphological changes (118, 119). Mutations within cells can be detected by the immune surveillance and initiate the immune response to eliminate “nonself” cells in the localized region where precancerous transformation occurs. In most cases, these cells are cleared by immune surveillance system. However, in immunosuppressive microenvironment where immune surveillance is diminished due to chronic inflammation or oncolytic virus, the infiltration of immune cells will decrease, and the cytokine interleukin-6 (IL-6) will activate the JAK/STAT3 signaling pathway in monocytes (120), which ultimately contributes to abnormal cells escaping immune elimination and proliferating into carcinoma in situ (CIS). The landscape of the TIME of CIS has been profiled, and the distribution and immune pattern of tumor-infiltrating immune cells are affected by the heterogeneity of tumor cells (121, 122). Therefore, the spatial distribution of immune components is involved in the transition of “normal” cells to tumor cells and CIS.

Knowledge about the spatial architecture of the TIME in the tumor initiation helps to reveal the evolutionary trajectory of premalignancy toward malignancy. However, conventional technologies have difficulties in detecting occult lesions, limiting the conduct of research (123, 124). It is believed that the availability of spatial information about the TIME expands the information dimension of precancer lesions, which can help researchers understand tumor initiation and guide clinical practice.

### Spatial architecture of the TIME in tumor expansion and invasion

Developing tumors are primary targets for research in spatial architecture of the TIME because of their appropriate size, enrichment in immune components, and the bidirectional roles of spatial architecture of the TIME within these tissues (Fig. 4b). Tumor expansion is accompanied by physiological processes, such as tumor cell proliferation, angiogenesis and immune cell infiltration. Description of the immune cell infiltration patterns based on spatial information provides additional insights into the tumor invasion. In some samples, immune cells are distributed diffusely, while in others, they tend to aggregate to form CNs, which are considered able to enhance immune cell function. In most cases, tumor-infiltrating immune cells are tumor-killing cells, and thus CN formation can fortify antitumor immunity. Nevertheless, some other CNs may consist of immunosuppressive cells, which reversely attenuate antitumor immunity (10, 19). The formation and impacts of those known structures on antitumor immunity are underexplored, and will probably be the focus of subsequent study.

Invasion, which is necessary for tumors to metastasize, occurs on the border of the tumor and is regulated by the frontier of the TIME. One of the most crucial events in tumor invasion is the epithelial–mesenchymal transition (EMT) (125). Studies of head and neck tumors identified a fraction of cells that were spatially located close to CAFs and immune cells in the leading edge of the tumor, expressing a signature related to a partial EMT program. This study reflected the relationship between the active immune response at the invasion margin and the occurrence of metastasis (126).

Except for EMT and partial EMT cells, previous studies have reported a higher abundance of immune cells and a stronger antitumor immune effect of the IM than the TC at metastatic lesions of gastroesophageal adenocarcinomas (127), hepatocellular carcinoma (128), melanoma (12), and colorectal cancer (129–131). This phenomenon may be driven by the intense struggle in the ecological niche between marginal tumor cells and immune cells, which is similar to that at the invading edge during ecological species invasion. Based on this analogy, the Darwinian dynamics model in ecology has been used to model the spatiotemporal distribution and evolutionary shifts of immune components at the invasion edge of the TIME in silico (132, 133).

### Spatial architecture of the TIME and metastases

After invasion, tumor cells traverse the stroma, enter the vascular system and colonize a secondary site, namely metastasis occurs. Current studies about the TIME’s spatial architecture in metastases mainly focus on specific spatial patterns (Fig. 4c).

As mentioned above, spatial patterns indicated the structure formed by well-organized TIME components. Representative spatial patterns in metastasis is the niche induced by exosomes and/or factors from the primary site (134). Before the seeding of circulating tumor cells (CTCs), the primary tumor-derived components facilitate the recruitment of immune cells into the niche, while immune cells subsequently, modify the local microenvironment of niches to form a feedback loop that ultimately promotes the formation of premetastatic niche (100, 135, 136). Specifically, premetastatic niches construct an immunosuppressive microenvironment composed of myeloid-derived suppressor cells (137), regulatory T cells (Tregs) (138), TAMs (139, 140), and tumor-associated neutrophils (141) to abolish local antitumor immunity that is more conducive to CTC colonization. The immunoregulatory components and their functions within niches have been thoroughly studied and recently reviewed (100, 142). Several studies have progressively revealed the spatial distribution of immune and host stromal components within niches (143–145).

After the seeding of CTCs, a premetastatic niche becomes a metastatic niche, which inherits the spatial architecture and functional status of the premetastatic niche, including emerging vessels, vascular leakiness and an immunosuppressive TIME. Moreover, immune components regulate spatiotemporal tumor cell stemness and plasticity (146, 147). Tumor cells also exert a spatially dependent effect on their surrounding cells (148). The formation and development of premetastatic niches and metastatic niches are still complex, and the evolution of these processes should be further explored.

Tumor cells in niches proliferate and ultimately grow into metastasis. Current investigations about the spatial heterogeneity of metastases tend to explore differences among primary sites and metastases in different sites, which expands the conception of spatial heterogeneity to a more macro level with greater clinical value. In addition to heterogeneity, studies have identified some consistencies between the spatial arrangement of immune cells in the TIME of metastatic and primary foci (149). For instance, in ovarian cancer, researchers found that subpopulations of T cells in primary tumors or different metastatic sites are relatively consistent, indicating their ability to track disseminated tumor cells through space (56, 106, 150–152). Meanwhile, no difference was observed in the distance between CD8^+^ T cells and tumor cells in primary and metastatic melanoma foci (12). The heterogeneity and consistency discussed above highlight the complexity of TIME’s spatial architecture, and more researches are required for clear elucidation.

## Spatially-based extracellular nonspecific chemical (ENSC) gradients orchestrate immune cell function

In addition to “tangible” structures in the TME, “intangible” extracellular nonspecific chemicals (ENSCs) are also critical components of the TME. In contrast to immune molecules, such as diverse interleukins (ILs), ENSCs refer to extracellular molecules participating in the metabolism of almost all cells and exerting extensive effects on them, including immune cells in the TIME. ENSCs manifest spatial heterogeneity in the form of “gradient”. And though ENSCs are not components of TIME, their unignorable role in the regulation of the spatial architecture of TIME emphasizes their importance in the research of TIME. In this section, we will discuss the current knowledge of the distribution of ENSCs in the TME and briefly review the effects of the ENSC distribution on immune cells.

### Basic principle of the distribution of ENSCs

Some basic physical and biological principles can help us to understand the distribution of ENSCs. ENSCs include a variety of chemicals, whose distribution is complex and interdependent. Depicting a general map of all ENSCs in the TME is infeasible and unnecessary because they vary among different species, tissue origins, patients, lesion locations, stages, and systemic conditions (28, 52). Nevertheless, the distribution of ENSCs is also spatially regulated due to some basic principles it must obey, which can help us grasp the outline of its landscape.

These basic principles are described below, and most of them are self-evident but essential.

1. The appearance and disappearance of ENSCs are in partial hemostasis and depend on two mechanisms, blood circulation and in situ metabolism, which are tightly interlaced.
2. Powered by the concentration gradient, ENSCs passively diffuse from areas of high density toward those of low density.
3. Competition exists between tumor cells and immune cells for most ENSCs, and tumor cells typically have an advantage (153, 154).
4. The distribution of ENSCs observed at a specific time point is a cross section of consecutive biological processes.

### Oxygen is pivotal for ENSCs

Oxygen is capable of being the hallmark molecule among all ENSCs. Various types of ENSCs are present in the TME, i.e., oxygen, glucose, carbon dioxide, lactate, amino acids, metal ions, lipids, etc. Considering the high heterogeneity and intricacy of ENSCs in the TME, a judicious approach is to select some representative ENSCs to better understand their distribution and biological effects. As far as we are concerned, oxygen should be critical for several reasons.

First, cellular respiration is the basis of cell metabolism and survival, in which oxygen is irreplaceable in the long term. The transportation and utilization of oxygen among most types of tumors are highly homogeneous, which dramatically facilitates associated studies. Meanwhile, the oxygen gradient serves as an ideal marker for the distance from vessels, and is closely related to other ENSCs by metabolism directly or indirectly. In addition, alterations in oxygenation and its subsequent outcomes are deeply incorporated into the biological transformation of tumors, such as angiogenesis, progression, and necrosis. Taken together, we choose oxygen as a representative ENSC when studying the spatial architecture of the TIME.

**Fig. 5:**
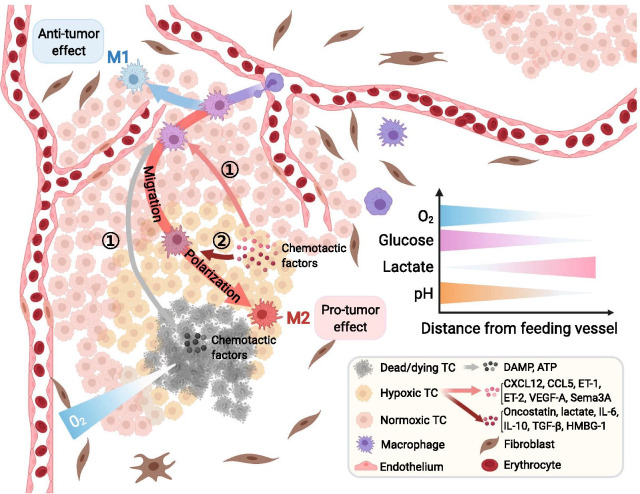
Oxygen serves as a pivotal extracellular nonspecific chemical (ENSC), and its gradient orchestrates the tumor immune microenvironment (TIME). The aberrant structure of tumor vessels and the abnormal distribution of oxygen in tumors feature a consecutive normoxia-hypoxia-anoxia gradient from feeding vessels toward the tumor center. Chemotactic factors such as CXCL12, CCL5, ET-1, ET-2, VEGF-A and Sema3A released by hypoxic tumor cells, as well as damage-associated molecular patterns (DAMPs) and ATP released by dead/dying tumor cells, attract macrophages to infiltrate into the hypoxic tumor core. Cytokines such as oncostatin, IL-6, IL-10, TGF-β, and HMBG-1 and lactate produced by hypoxic tumor cells further promote the differentiation of macrophages into protumor M2 macrophages, while macrophages remaining next to normoxic feeding vessels display an antitumor phenotype. The oxygen gradient may serve as a marker of the distance from feeding vessels and correlates with other ENSC gradients in the TIME, such as glucose, lactate and hydrion. CXCL12, C-X-C chemokine ligand type 12; CCL5, C–C chemokine ligand type 5; ET, endothelin; VEGF, vascular endothelial growth factor; IL, interleukin; TC, tumor cell; TGF, transforming growth factor; HMBG-1, high mobility group box 1 protein;Sema3A, semaphorin-3A

The oxygen distribution in normal tissue is regulated by an exquisite mechanism to form balanced dynamic homeostasis, but processes occurring in tumors are aberrant (155–157). The rapid proliferation of tumor cells exceeds the oxygen and nutrient supply and subsequently leads to angiogenesis. Neovascularization, characterized by an abnormal vessel wall structure with unbalanced distribution and immature function, causes an insufficient and unbalanced oxygen supply (158–160). These factors all contribute to the abnormal distribution of oxygen, featuring a consecutive normoxia-hypoxia-anoxia gradient from feeding vessels toward the tumor center, which has been certified in vivo, in vitro and in silico (159, 161–165) (Fig. 5).

The gradient of oxygen in the TME exerts comprehensive effects on the biological performance of diverse immune cells, including infiltration, migration, polarization, function, and metabolism (166). Here, we use macrophages, one of the most well-studied immune cells in this field, as an example.

Macrophages are innate immune cells residing in all tissues derived from monocytes in blood. M1 macrophages, a subset of macrophages displaying an antitumor phenotype, are more commonly located in normoxic areas close to feeding vessels, while M2 macrophages, the protumor subset, are more dominant in hypoxic TCs (167). Although in vitro experiments have correlated the polarization of macrophages with hypoxia inducible factor (HIF)-1 and HIF-2 (168), in vivo research revealed that hypoxia does not directly affect the activation of macrophages but exerts its effect through hypoxic tumor cells (167, 169, 170). The migration of macrophages from the TS toward the TC is mediated by the interaction between receptors on macrophages and chemotactic molecules released by tumor cells. In the TC, which lacks oxygen, dying tumor cells release damage-associated molecular patterns (DAMPs) and adenosine triphosphate (ATP). Hypoxic tumor cells around dying tumor cells in the TC can also produce various kinds of cytokines regulated by HIF, such as C–C chemokine ligand type 5 (CCL5), CXCL12, vascular endothelial growth factor A (VEGF-A), endothelin (ET)-1, ET-2 and semaphorin-3A (Sema3A). All these molecules bind to receptors primarily regulated by HIF on macrophages, which promotes the migration of macrophages toward the TC (166). Then, polarization toward M2 macrophages occurs with the intervention of lactate, IL-4, transforming TGF-β, oncostatin, eotaxin, and other molecules secreted by hypoxic tumor cells. In summary, the function of macrophages is closely related to the intratumor spatial heterogeneity of oxygenation.

The functions of other immune cells are also correlated with the oxygen gradient, such as the migration of neutrophils, natural killer (NK) cells, and Tregs toward hypoxic regions and the stagnancy of CD4^+^ T cells and CD8^+^ T cells in normoxic areas. The corresponding retention or alteration of their functions has been elaborated previously (166, 171–173). Nevertheless, the relations of various immune cells with the oxygenation spatial gradient still need further exploration.

### The distribution of other ENSCs in the TME and their effect on immune cells

The distributions of other ENSCs (or their uptake) have also been partially revealed in vivo or in vitro to varying degrees, such as glutamine, amino acids, and metal ions (165, 174–177). The distributions of some of these molecules are correlated with the function of immune cells. For instance, the altered gradient of Na^+^ is correlated with the polarization of macrophages, probably in a tissue-specific manner (176), and increased K^+^ levels in tumors can suppress effector T cell function and prevent immune cells from maturing (177). However, researchers still do not comprehensively understand the significance of their effects on immune cells in a complex real TME, which must be further established.

Recently, tumor metabolomics has attracted increasing interest from researchers. Using high-flux tools to reveal the metabolism of tissue and cells, a series of metabolites were found to play an important role in the interaction between tumor cells and immune cells (178, 179). With further investigations of their spatial distribution, additional mechanisms and potential therapeutic targets might be proposed.

## Spatial architecture of the TIME in clinical application

The clinical application of spatial architecture of the TIME can be roughly divided into two aspects, prognosis prediction and clinical treatment.

### Spatial architecture of the TIME in prognosis prediction

Many prognostic implications of compartment-based features have been described. The Immunoscore is a simplified tool based on the immune context that is used to evaluate the abundance of T cell populations in the TC or IM jointly and shows great clinical potential in colorectal cancer (180–183). Apart from the Immunoscore, other researchers also examined the distribution of some specific subtypes of immune cells in different compartments within the TME. The specific distributions of CD8^+^ T cells and CD163^+^ macrophages in breast cancer (184), cytotoxic T cells (CTLs) and memory T cells in colorectal cancer (185), CD8^+^ T cells and Tregs in NSCLC (186), and CTLs in primary melanoma (81) residing in different compartments were found to be significantly related to clinical outcomes, improving our knowledge of a precise diagnosis.

Diverse parameters focusing on  the distances of different cell–cell pairs are also well established. In breast cancer, patients with a mixed distribution of tumor and immune cells experienced prolonged survival compared with those whose tumor cells and immune cells were segregated from each other, even if the latter may have more abundant immune cells (19). Quantitative research found that enriched CD8^+^ cells within distant stroma (farther than one tumor cell diameter away from the TC) rather than adjacent stroma (within one tumor cell diameter) were related to prolonged disease-specific survival in patients with breast carcinoma (187). Mezheyeuski et al. (188) calculated the shortest distance from each immune cell to the nearest neighboring cancer cell and discovered that the spatial proximity of Tregs and tumor cells was correlated with a significantly unfavorable survival in patients with NSCLC. Nearchou et al. (79) analyzed the number of CD8^+^ T cells within 100 μm radii from tumor buds and found that their totality and distance were significantly associated with disease-specific survival. Enfield et al. (189) discovered that the frequencies of CD3^+^CD8^+^ T cells around tumor cells were a more powerful marker to predict low recurrence than their density in lung adenocarcinoma. For spatial architecture of the TIME based on immune targets, researches are relatively limited. Lazarus et al. revealed that CTLs were more abundant around PD-L1^−^ epithelial cells than PD-L1^+^ epithelial cells, and the engagement between CTLs and epithelial cells was correlated with favorable overall survival (106).

As for patterned structures of the TIME, clinical data showed that the existence of TLSs is significantly related to improved overall survival (OS), disease-specific survival (DSS) and disease-free survival (DFS) in patients with early stage NSCLC (190). Further studies found that TLSs are associated with increased lymphocyte infiltration into tumors with extended DFS in breast cancer and colorectal cancer (191). However, the role of TLSs in predicting the prognosis is still under debate because another study of breast cancer showed that patients with abundant TLSs exhibited worse DFS and OS than those lacking TLSs (192). The cellular component in TLSs has also aroused the interest of scientists. One study found that TLSs in patients with spontaneous prostate tumor regression contain fewer Tregs and more T-helper 1 (Th1) and CD8^+^ T cells, which are commonly considered antitumor components (193). The detection of premetastatic and metastatic niches in the early stage of metastasis may facilitate effective treatment shortly after or even before the seeding of CTCs. Their existence can be determined by changes in the histological tissue density of potential metastatic sites (194, 195) or biochemical/cellular biomarkers in circulation (137, 196, 197). These discoveries might indicate unfavorable events prior to clinical manifestation and help clinicians provide early treatment.

### Spatial architecture of the TIME in clinical treatment

To remodeling the spatial architecture of the TIME, diverse therapeutic strategies have also been proposed, including those that promote (116, 198) or prevent (199) immune cells from infiltrating into designated areas of the TIME, modify angiogenesis or oxygenation of the TIME (200–202), induce the formation of TLSs (85, 86) or modulate members of TLSs (87), regulate stromal components in the TIME (203, 204), and transform ENSCs in the TIME (178, 205). Regretfully, there are few researches focusing on the value of the spatial architecture of the TIME in treatment response prediction.

Profound mechanisms likely underlie the correlation between spatial features and clinical outcomes, which remain to be fully revealed for researchers to design more feasible therapies that will become available for patients in the future.

## Conclusions and perspectives

In this article, we provide a systematic summary of technological advances and related researches on the spatial architecture of the TIME. Recently, an explosion of researches witnessed profound advances in high-dimensional techniques that preserve the spatial information, which has revolutionized our understanding about the spatial architecture of the TIME.

Despite the recent rapid development, there are still many unresolved issues in the study of TIME’s spatial architecture. First of all, spatial proximity between cells does not necessarily mean that actual interactions are occurring. Current analyses assume that cells in close proximity have a higher probability of potential interaction, but whether an interaction exists and its extent must be further verified. The in vitro study of cell-to-cell interactions is feasibly facilitated by cell culture techniques, such as coculture and organoids (206). However, these models do not fully reflect the situation in vivo. The mCherry niche labeling strategy devised by Ombrato et al. showed the in vivo intercellular material flow of tumor cells and their neighborhood in metastatic niches through the secretion and uptake of marker proteins. However, due to the disparity in the phagocytic capacity of different neighboring immune cells themselves, the simple secretion-uptake model does not fully reflect interactions within the TIME (148). Therefore, future in-depth studies of in situ spatial interactions and space-dependent functions will depend on the further development of in vivo and in vitro models. Moreover, only limited studies have revealed mechanisms underlying the formation, development and regulation of spatial architecture of the TIME, partially due to the lack of suitable experimental models.

Conventionally, in vitro experiments are widely favored because of their controllability and ease of use. Experimental techniques, such as extracellular matrix culturing cancer cells in biomimetic scaffolds (207), reproduce the spatial distribution of tumor cells in artificially constructed meshwork in vitro to some extent. However currently, immune cells cannot be cultured in scaffolds due to the difficulty in culturing immune cells and their short survival time in vitro. Organoids and spheroids are popular platforms adopted in the investigation of tumors recently, which can partially preserve the spatial information of original malignancies. Typically, immune cells are not included in organoids or spheroids. Novel strategies such as the air–liquid interface have been proposed to introduce immune cells into these structures, and their clinical potential has been approved (208–210). The technological modification for the culturing and editing of organoids and spheroids might bring us to a new era of the investigation of the TIME. Recent studies about the TIME rely more on transplanted tumors as well as patient-derived xenograft. Although in vivo models can relatively better reflect the real situation, the uncontrollability of animals increases the technical difficulty and confounding of the experiment (211). We hope that in the future, the complex structural networks of the TIME will be constructed in robust experimental models.

In addition, the majority of existing studies on spatial architecture of the TIME are descriptive, with most samples obtained from retrospective cross-sectional studies. We suggest analyzing the spatial architecture of the TIME in terms of temporal dynamics. For example, with superresolution intravital fluorescence microscopy, a prospective study can be carried out to investigate T cell behavior within specimens collected before and after immunotherapy to identify spatial structural changes (212). The description and tracking of the spatiotemporal heterogeneity and evolution of the TIME will provide additional insights into tumor diagnosis and treatment.

**Fig. 6:**
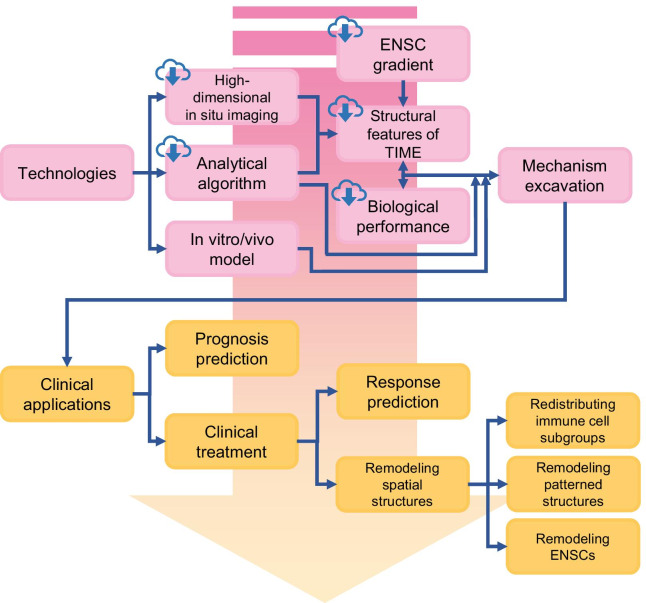
Future development of the tumor immune microenvironment. The development of technologies in high-dimensional in situ imaging, analytical algorithms and in vitro/*vivo* models will promote the further elucidation of mechanisms underlying the tumor immune microenvironment (TIME) (boxes with cloud marks refer to content that future online databases of TIME might include). Profound advances in the clinical application of TIME rely on deeper insights into the TIME, which will help physicians with determining both a precise diagnosis and therapy

In summary, we consider the spatial architecture of the TIME a promising direction for academic research and clinical application in this field (Fig. 6). As an emerging field, the spatial architecture of the TIME has a large number of unknowns waiting to be explored. With the foreseeable demand for a more comprehensive landscape of the spatial architecture of the TIME, the near future will probably witness newer technologies with better precision, faster scanning, more convenient protocols, lower cost, and public online archives. Improved technologies facilitating sharing and robust softwares or algorithms to query massive databases will also increase accessibility for other researchers in this field.

## Data Availability

Not applicable.

## Abbreviations

CAFs: Cancer-associated fibroblasts
CIS: Carcinoma in situ
CNN: Convolutional neural network
CNs: Cellular neighborhoods
CTCs: Circulating tumor cells
CTLs: Cytotoxic T cells
DFS: Disease-free survival
EMT: Epithelial–mesenchymal transition
ENSCs: Extracellular nonspecific chemicals
FFPE: Formalin-fixed and paraffin-embedded
FISH: Fluorescence in situ hybridization
H&E staining: Hematoxylin and eosin staining
HIF: Hypoxia inducible factor
IDO: Indoleamine2, 3-dioxygenase
IHC: Immunohistochemistry
IM: Invasive margin
NSCLC: Non-small cell lung cancer
OS: Overall survival
PD-1: Programmed cell death 1
scRNA-seq: Single-cell RNA sequencing
TAMs: Tumor-associated macrophages
TC: Tumor core
TIME: Tumor immune microenvironment
TLSs: Tertiary lymphoid structures
Tregs: Regulatory T cells
TS: Tumor stoma

## References

[1] Hanahan D, Weinberg RA (2011). Hallmarks of cancer: the next generation. Cell.

[2] Anderson NM, Simon MC (2020). The tumor microenvironment. Curr Biol.

[3] Riera-Domingo C, Audige A, Granja S, Cheng WC, Ho PC, Baltazar F (2020). Immunity, hypoxia, and metabolism-the menage a trois of cancer: implications for immunotherapy. Physiol Rev.

[4] Gajewski TF, Schreiber H, Fu YX (2013). Innate and adaptive immune cells in the tumor microenvironment. Nat Immunol.

[5] Bader JE, Voss K, Rathmell JC (2020). Targeting metabolism to improve the tumor microenvironment for cancer immunotherapy. Mol Cell.

[6] Binnewies M, Roberts EW, Kersten K, Chan V, Fearon DF, Merad M (2018). Understanding the tumor immune microenvironment (TIME) for effective therapy. Nat Med.

[7] Greten FR, Grivennikov SI (2019). Inflammation and cancer: triggers, mechanisms, and consequences. Immunity.

[8] Pitt JM, Marabelle A, Eggermont A, Soria JC, Kroemer G, Zitvogel L (2016). Targeting the tumor microenvironment: removing obstruction to anticancer immune responses and immunotherapy. Ann Oncol.

[9] Zemek RM, De Jong E, Chin WL, Schuster IS, Fear VS, Casey TH, et al. Sensitization to immune checkpoint blockade through activation of a STAT1/NK axis in the tumor microenvironment. Sci Transl Med. 2019;11(501). 10.1126/scitranslmed.aav7816 31316010

[10] Schürch C, Bhate S, Barlow G, Phillips D, Noti L, Zlobec I, et al. Coordinated cellular neighborhoods orchestrate antitumoral immunity at the colorectal cancer invasive front. Cell. 2020. 10.1016/j.cell.2020.10.021 PMC7658307 33125896

[11] Casasent AK, Schalck A, Gao R, Sei E, Long A, Pangburn W (2018). Multiclonal invasion in breast tumors identified by topographic single cell sequencing. Cell.

[12] Gide TN, Silva IP, Quek C, Ahmed T, Menzies AM, Carlino MS (2020). Close proximity of immune and tumor cells underlies response to anti-PD-1 based therapies in metastatic melanoma patients. Oncoimmunology.

[13] Zhang L, Conejo-Garcia JR, Katsaros D, Gimotty PA, Massobrio M, Regnani G (2003). Intratumoral T cells, recurrence, and survival in epithelial ovarian cancer. N Engl J Med.

[14] Fridman WH, Galon J, Pagès F, Tartour E, Sautès-Fridman C, Kroemer G (2011). Prognostic and predictive impact of intra- and peritumoral immune infiltrates. Cancer Res.

[15] Ali HR, Jackson HW, Zanotelli VRT, Danenberg E, Fischer JR, Bardwell H (2020). Imaging mass cytometry and multiplatform genomics define the phenogenomic landscape of breast cancer. Nat Cancer.

[16] Jackson HW, Fischer JR, Zanotelli VRT, Ali HR, Mechera R, Soysal SD (2020). The single-cell pathology landscape of breast cancer. Nature.

[17] Arnol D, Schapiro D, Bodenmiller B, Saez-Rodriguez J, Stegle O (2019). Modeling cell-cell interactions from spatial molecular data with spatial variance component analysis. Cell Rep.

[18] Stoltzfus CR, Filipek J, Gern BH, Olin BE, Leal JM, Wu Y (2020). CytoMAP: a spatial analysis toolbox reveals features of myeloid cell organization in lymphoid tissues. Cell Rep.

[19] Keren L, Bosse M, Marquez D, Angoshtari R, Jain S, Varma S (2018). A structured tumor-immune microenvironment in triple negative breast cancer revealed by multiplexed ion beam imaging. Cell.

[20] Zhu Q, Shah S, Dries R, Cai L, Yuan G-C (2018). Identification of spatially associated subpopulations by combining scRNAseq and sequential fluorescence in situ hybridization data. Nat Biotechnol.

[21] Schapiro D, Jackson HW, Raghuraman S, Fischer JR, Zanotelli VRT, Schulz D (2017). histoCAT: analysis of cell phenotypes and interactions in multiplex image cytometry data. Nat Methods.

[22] Turkki R, Linder N, Kovanen PE, Pellinen T, Lundin J (2016). Antibody-supervised deep learning for quantification of tumor-infiltrating immune cells in hematoxylin and eosin stained breast cancer samples. J Pathol Inform.

[23] Saltz J, Gupta R, Hou L, Kurc T, Singh P, Nguyen V (2018). Spatial organization and molecular correlation of tumor-infiltrating lymphocytes using deep learning on pathology images. Cell Rep.

[24] Linder N, Taylor JC, Colling R, Pell R, Alveyn E, Joseph J (2019). Deep learning for detecting tumour-infiltrating lymphocytes in testicular germ cell tumours. J Clin Pathol.

[25] Fu Y, Jung AW, Torne RV, Gonzalez S, Vöhringer H, Shmatko A (2020). Pan-cancer computational histopathology reveals mutations, tumor composition and prognosis. Nat Cancer.

[26] Liu Y, Li X, Zheng A, Zhu X, Liu S, Hu M (2020). Predict Ki-67 positive cells in H&E-stained images using deep learning independently from IHC-stained images. Front Mol Biosci.

[27] Levsky JM, Singer RH (2003). Fluorescence in situ hybridization: past, present and future. J Cell Sci.

[28] Goltsev Y, Samusik N, Kennedy-Darling J, Bhate S, Hale M, Vazquez G (2018). Deep profiling of mouse splenic architecture with CODEX multiplexed imaging. Cell.

[29] Coskun AF, Cai L (2016). Dense transcript profiling in single cells by image correlation decoding. Nat Methods.

[30] Eng CL, Lawson M, Zhu Q, Dries R, Koulena N, Takei Y (2019). Transcriptome-scale super-resolved imaging in tissues by RNA seqFISH. Nature.

[31] Lubeck E, Coskun AF, Zhiyentayev T, Ahmad M, Cai L (2014). Single-cell in situ RNA profiling by sequential hybridization. Nat Methods.

[32] Keren L, Bosse M, Thompson S, Risom T, Vijayaragavan K, McCaffrey E (2019). MIBI-TOF: A multiplexed imaging platform relates cellular phenotypes and tissue structure. Sci Adv.

[33] Angelo M, Bendall SC, Finck R, Hale MB, Hitzman C, Borowsky AD (2014). Multiplexed ion beam imaging of human breast tumors. Nat Med.

[34] Giesen C, Wang HA, Schapiro D, Zivanovic N, Jacobs A, Hattendorf B (2014). Highly multiplexed imaging of tumor tissues with subcellular resolution by mass cytometry. Nat Methods.

[35] Chang Q, Ornatsky OI, Siddiqui I, Loboda A, Baranov VI, Hedley DW (2017). Imaging mass cytometry. Cytometry A J Int Soc Anal Cytol.

[36] Satija R, Farrell JA, Gennert D, Schier AF, Regev A (2015). Spatial reconstruction of single-cell gene expression data. Nat Biotechnol.

[37] Moncada R, Barkley D, Wagner F, Chiodin M, Devlin JC, Baron M (2020). Integrating microarray-based spatial transcriptomics and single-cell RNA-seq reveals tissue architecture in pancreatic ductal adenocarcinomas. Nat Biotechnol.

[38] Hu KH, Eichorst JP, McGinnis CS, Patterson DM, Chow ED, Kersten K (2020). ZipSeq: barcoding for real-time mapping of single cell transcriptomes. Nat Methods.

[39] Crecelius AC, Schubert US, von Eggeling F (2015). MALDI mass spectrometric imaging meets "omics": recent advances in the fruitful marriage. Analyst.

[40] Ryan DJ, Spraggins JM, Caprioli RM (2019). Protein identification strategies in MALDI imaging mass spectrometry: a brief review. Curr Opin Chem Biol.

[41] xxx

[42] Andrés-Manzano MJ, Andrés V, Dorado B (2015). Oil red O and hematoxylin and eosin staining for quantification of atherosclerosis burden in mouse aorta and aortic root. Methods Mol Biol (Clifton, NJ).

[43] Cardiff RD, Miller CH, Munn RJ (2014). Manual hematoxylin and eosin staining of mouse tissue sections. Cold Spring Harb Protoc.

[44] Cottrell TR, Thompson ED, Forde PM, Stein JE, Duffield AS, Anagnostou V (2018). Pathologic features of response to neoadjuvant anti-PD-1 in resected non-small-cell lung carcinoma: a proposal for quantitative immune-related pathologic response criteria (irPRC). Ann Oncol.

[45] Komura D, Ishikawa S (2019). Machine learning approaches for pathologic diagnosis. Virchows Archiv Int J Pathol.

[46] De Logu F, Ugolini F, Maio V, Simi S, Cossu A, Massi D (2020). Recognition of cutaneous melanoma on digitized histopathological slides via artificial intelligence algorithm. Front Oncol.

[47] Ha EJ, Baek JH, Na DG (2019). Deep convolutional neural network models for the diagnosis of thyroid cancer. Lancet Oncol.

[48] Wollmann T, Gunkel M, Chung I, Erfle H, Rippe K, Rohr K (2019). GRUU-Net: integrated convolutional and gated recurrent neural network for cell segmentation. Med Image Anal.

[49] Sim Y, Chung MJ, Kotter E, Yune S, Kim M, Do S (2020). Deep convolutional neural network-based software improves radiologist detection of malignant lung nodules on chest radiographs. Radiology.

[50] Lian C, Liu M, Zhang J, Shen D (2020). Hierarchical fully convolutional network for joint atrophy localization and Alzheimer's disease diagnosis using structural MRI. IEEE Trans Pattern Anal Mach Intell.

[51] Wood KJ, Bushell A, Hester J (2012). Regulatory immune cells in transplantation. Nat Rev Immunol.

[52] Faget J, Groeneveld S, Boivin G, Sankar M, Zangger N, Garcia M (2017). Neutrophils and snail orchestrate the establishment of a pro-tumor microenvironment in lung cancer. Cell Rep.

[53] Domon B, Aebersold R (2006). Mass spectrometry and protein analysis. Science.

[54] The path of biomolecular mass spectrometry into open research. Nat Commun. 2019;10(1):4029. 10.1038/s41467-019-12150-4 PMC6733844 31501439

[55] Ståhl PL, Salmén F, Vickovic S, Lundmark A, Navarro JF, Magnusson J (2016). Visualization and analysis of gene expression in tissue sections by spatial transcriptomics. Science.

[56] Thrane K, Eriksson H, Maaskola J, Hansson J, Lundeberg J (2018). Spatially resolved transcriptomics enables dissection of genetic heterogeneity in stage III cutaneous malignant melanoma. Cancer Res.

[57] Berglund E, Maaskola J, Schultz N, Friedrich S, Marklund M, Bergenstrahle J (2018). Spatial maps of prostate cancer transcriptomes reveal an unexplored landscape of heterogeneity. Nat Commun.

[58] Villacampa EG, Larsson L, Kvastad L, Andersson A, Carlson J, Lundeberg J. Genome-wide Spatial Expression Profiling in FFPE Tissues. BioRxiv. 2020.

[59] Gruosso T, Gigoux M, Manem VSK, Bertos N, Zuo D, Perlitch I (2019). Spatially distinct tumor immune microenvironments stratify triple-negative breast cancers. J Clin Invest.

[60] Halama N, Michel S, Kloor M, Zoernig I, Benner A, Spille A (2011). Localization and density of immune cells in the invasive margin of human colorectal cancer liver metastases are prognostic for response to chemotherapy. Cancer Res.

[61] Laghi L, Bianchi P, Miranda E, Balladore E, Pacetti V, Grizzi F (2009). CD3+ cells at the invasive margin of deeply invading (pT3-T4) colorectal cancer and risk of post-surgical metastasis: a longitudinal study. Lancet Oncol.

[62] Roesch A, Vogt T, Stolz W, Dugas M, Landthaler M, Becker B (2003). Discrimination between gene expression patterns in the invasive margin and the tumour core of malignant melanomas. Melanoma Res.

[63] Phanthunane C, Wijers R, de Herdt M, Langeveld TPM, Koljenovic S, Dasgupta S (2021). B-cell clusters at the invasive margin associate with longer survival in early-stage oral-tongue cancer patients. Oncoimmunology.

[64] Glajcar A, Szpor J, Pacek A, Tyrak KE, Chan F, Streb J (2017). The relationship between breast cancer molecular subtypes and mast cell populations in tumor microenvironment. Virchows Archiv Int J Pathol.

[65] Lara OD, Krishnan S, Wang Z, Corvigno S, Zhong Y, Lyons Y (2019). Tumor core biopsies adequately represent immune microenvironment of high-grade serous carcinoma. Sci Rep.

[66] Liu F, Liu W, Zhou S, Yang C, Tian M, Jia G, et al. Identification of FABP5 as an immunometabolic marker in human hepatocellular carcinoma. J Immunother Cancer. 2020;8(2). 10.1136/jitc-2019-000501 PMC7332195 32611686

[67] Galon J, Costes A, Sanchez-Cabo F, Kirilovsky A, Mlecnik B, Lagorce-Pagès C (2006). Type, density, and location of immune cells within human colorectal tumors predict clinical outcome. Science.

[68] Zeiner PS, Preusse C, Golebiewska A, Zinke J, Iriondo A, Muller A (2019). Distribution and prognostic impact of microglia/macrophage subpopulations in gliomas. Brain Pathol (Zurich, Switzerland).

[69] Halle S, Halle O, Förster R (2017). Mechanisms and dynamics of T cell-mediated cytotoxicity in vivo. Trends Immunol.

[70] de la Iglesia JV, Slebos RJC, Martin-Gomez L, Wang X, Teer JK, Tan AC (2020). Effects of tobacco smoking on the tumor immune microenvironment in head and neck squamous cell carcinoma. Clin Cancer Res.

[71] Kim HD, Kim JH, Ryu YM, Kim D, Lee S, Shin J, et al. Spatial distribution and prognostic implications of tumor-infiltrating FoxP3- CD4+ T cells in biliary tract cancer. Cancer Res Treat. 2020. 10.4143/crt.2020.704 PMC7812013 32878426

[72] Liu M, Kuo F, Capistrano KJ, Kang D, Nixon BG, Shi W (2020). TGF-β suppresses type 2 immunity to cancer. Nature.

[73] Joshi K, de Massy MR, Ismail M, Reading JL, Uddin I, Woolston A (2019). Spatial heterogeneity of the T cell receptor repertoire reflects the mutational landscape in lung cancer. Nat Med.

[74] Okrah K, Tarighat S, Liu B, Koeppen H, Wagle MC, Cheng G (2018). Transcriptomic analysis of hepatocellular carcinoma reveals molecular features of disease progression and tumor immune biology. NPJ Precis Oncol.

[75] Zheng B, Wang D, Qiu X, Luo G, Wu T, Yang S (2020). Trajectory and functional analysis of PD-1(high) CD4(+)CD8(+) T cells in hepatocellular carcinoma by single-cell cytometry and transcriptome sequencing. Adv Sci (Weinheim, Baden-Wurttemberg, Germany).

[76] Schwen LO, Andersson E, Korski K, Weiss N, Haase S, Gaire F (2018). Data-driven discovery of immune contexture biomarkers. Front Oncol.

[77] Lundgren S, Elebro J, Heby M, Nodin B, Leandersson K, Micke P (2020). Quantitative, qualitative and spatial analysis of lymphocyte infiltration in periampullary and pancreatic adenocarcinoma. Int J Cancer.

[78] Berthel A, Zoernig I, Valous NA, Kahlert C, Klupp F, Ulrich A (2017). Detailed resolution analysis reveals spatial T cell heterogeneity in the invasive margin of colorectal cancer liver metastases associated with improved survival. Oncoimmunology.

[79] Nearchou IP, Lillard K, Gavriel CG, Ueno H, Harrison DJ, Caie PD (2019). Automated analysis of lymphocytic infiltration, tumor budding, and their spatial relationship improves prognostic accuracy in colorectal cancer. Cancer Immunol Res.

[80] Nagl S, Haas M, Lahmer G, Büttner-Herold M, Grabenbauer GG, Fietkau R (2016). Cell-to-cell distances between tumor-infiltrating inflammatory cells have the potential to distinguish functionally active from suppressed inflammatory cells. Oncoimmunology..

[81] Gartrell RD, Marks DK, Hart TD, Li G, Davari DR, Wu A (2018). Quantitative analysis of immune infiltrates in primary melanoma. Cancer Immunol Res.

[82] Rasmusson A, Zilenaite D, Nestarenkaite A, Augulis R, Laurinaviciene A, Ostapenko V (2020). Immunogradient indicators for antitumor response assessment by automated tumor-stroma interface zone detection. Am J Pathol.

[83] Feichtenbeiner A, Haas M, Büttner M, Grabenbauer GG, Fietkau R, Distel LV (2014). Critical role of spatial interaction between CD8^+^ and Foxp3^+^ cells in human gastric cancer: the distance matters. Cancer Immunol Immunother.

[84] Halse H, Colebatch AJ, Petrone P, Henderson MA, Mills JK, Snow H (2018). Multiplex immunohistochemistry accurately defines the immune context of metastatic melanoma. Sci Rep.

[85] Sautès-Fridman C, Petitprez F, Calderaro J, Fridman WH (2019). Tertiary lymphoid structures in the era of cancer immunotherapy. Nat Rev Cancer.

[86] Cabrita R, Lauss M, Sanna A, Donia M, Skaarup Larsen M, Mitra S (2020). Tertiary lymphoid structures improve immunotherapy and survival in melanoma. Nature.

[87] Helmink BA, Reddy SM, Gao J, Zhang S, Basar R, Thakur R (2020). B cells and tertiary lymphoid structures promote immunotherapy response. Nature.

[88] Petitprez F, de Reyniès A, Keung EZ, Chen TW, Sun CM, Calderaro J (2020). B cells are associated with survival and immunotherapy response in sarcoma. Nature.

[89] Munn LL, Jain RK (2019). Vascular regulation of antitumor immunity. Science.

[90] Beck B, Driessens G, Goossens S, Youssef KK, Kuchnio A, Caauwe A (2011). A vascular niche and a VEGF-Nrp1 loop regulate the initiation and stemness of skin tumours. Nature.

[91] Pietras A, Katz AM, Ekström EJ, Wee B, Halliday JJ, Pitter KL (2014). Osteopontin-CD44 signaling in the glioma perivascular niche enhances cancer stem cell phenotypes and promotes aggressive tumor growth. Cell Stem Cell.

[92] Hayakawa Y, Ariyama H, Stancikova J, Sakitani K, Asfaha S, Renz BW (2015). Mist1 expressing gastric stem cells maintain the normal and neoplastic gastric epithelium and are supported by a perivascular stem cell niche. Cancer Cell.

[93] Ghajar CM, Peinado H, Mori H, Matei IR, Evason KJ, Brazier H (2013). The perivascular niche regulates breast tumour dormancy. Nat Cell Biol.

[94] Murgai M, Ju W, Eason M, Kline J, Beury DW, Kaczanowska S (2017). KLF4-dependent perivascular cell plasticity mediates pre-metastatic niche formation and metastasis. Nat Med.

[95] Carlson P, Dasgupta A, Grzelak CA, Kim J, Barrett A, Coleman IM (2019). Targeting the perivascular niche sensitizes disseminated tumour cells to chemotherapy. Nat Cell Biol.

[96] Chen L, Oke T, Siegel N, Cojocaru G, Tam AJ, Blosser RL (2020). The immunosuppressive niche of soft-tissue sarcomas is sustained by tumor-associated macrophages and characterized by intratumoral tertiary lymphoid structures. Clin Cancer Res.

[97] Wang Q, He Z, Huang M, Liu T, Wang Y, Xu H (2018). Vascular niche IL-6 induces alternative macrophage activation in glioblastoma through HIF-2α. Nat Commun.

[98] Hughes R, Qian BZ, Rowan C, Muthana M, Keklikoglou I, Olson OC (2015). Perivascular M2 macrophages stimulate tumor relapse after chemotherapy. Cancer Res.

[99] Chen Z, Feng X, Herting CJ, Garcia VA, Nie K, Pong WW (2017). Cellular and molecular identity of tumor-associated macrophages in glioblastoma. Cancer Res.

[100] Liu Y, Cao X (2016). Characteristics and significance of the pre-metastatic niche. Cancer Cell.

[101] Prager BC, Xie Q, Bao S, Rich JN (2019). Cancer stem cells: the architects of the tumor ecosystem. Cell Stem Cell.

[102] Egen JG, Ouyang W, Wu LC (2020). Human anti-tumor immunity: insights from immunotherapy clinical trials. Immunity.

[103] Datar I, Sanmamed MF, Wang J, Henick BS, Choi J, Badri T (2019). Expression analysis and significance of PD-1, LAG-3, and TIM-3 in human non-small cell lung cancer using spatially resolved and multiparametric single-cell analysis. Clin Cancer Res.

[104] Villarroel-Espindola F, Yu X, Datar I, Mani N, Sanmamed M, Velcheti V (2018). Spatially resolved and quantitative analysis of VISTA/PD-1H as a novel immunotherapy target in human non-small cell lung cancer. Clin Cancer Res.

[105] Johnson DB, Bordeaux J, Kim JY, Vaupel C, Rimm DL, Ho TH (2018). Quantitative spatial profiling of PD-1/PD-L1 interaction and HLA-DR/IDO-1 predicts improved outcomes of anti-PD-1 therapies in metastatic melanoma. Clin Cancer Res.

[106] Lazarus J, Maj T, Smith JJ, Perusina Lanfranca M, Rao A, D'Angelica MI, et al. Spatial and phenotypic immune profiling of metastatic colon cancer. JCI Insight. 2018;3(22). 10.1172/jci.insight.121932 PMC6302940 30429368

[107] Coy S, Rashid R, Lin JR, Du Z, Donson AM, Hankinson TC (2018). Multiplexed immunofluorescence reveals potential PD-1/PD-L1 pathway vulnerabilities in craniopharyngioma. Neuro Oncol.

[108] Mansfield AS, Aubry MC, Moser JC, Harrington SM, Dronca RS, Park SS (2016). Temporal and spatial discordance of programmed cell death-ligand 1 expression and lymphocyte tumor infiltration between paired primary lesions and brain metastases in lung cancer. Ann Oncol.

[109] Zhang X, Cheng C, Hou J, Qi X, Wang X, Han P (2019). Distinct contribution of PD-L1 suppression by spatial expression of PD-L1 on tumor and non-tumor cells. Cell Mol Immunol.

[110] Giraldo NA, Nguyen P, Engle EL, Kaunitz GJ, Cottrell TR, Berry S (2018). Multidimensional, quantitative assessment of PD-1/PD-L1 expression in patients with Merkel cell carcinoma and association with response to pembrolizumab. J Immunother Cancer.

[111] Tsakiroglou AM, Fergie M, Oguejiofor K, Linton K, Thomson D, Stern PL (2020). Spatial proximity between T and PD-L1 expressing cells as a prognostic biomarker for oropharyngeal squamous cell carcinoma. Br J Cancer.

[112] Meurette O, Mehlen P (2018). Notch signaling in the tumor microenvironment. Cancer Cell.

[113] Fu Y, Liu S, Zeng S, Shen H (2018). The critical roles of activated stellate cells-mediated paracrine signaling, metabolism and onco-immunology in pancreatic ductal adenocarcinoma. Mol Cancer.

[114] Froeling FE, Feig C, Chelala C, Dobson R, Mein CE, Tuveson DA (2011). Retinoic acid-induced pancreatic stellate cell quiescence reduces paracrine Wnt-beta-catenin signaling to slow tumor progression. Gastroenterology.

[115] Johnson DE, O'Keefe RA, Grandis JR (2018). Targeting the IL-6/JAK/STAT3 signalling axis in cancer. Nat Rev Clin Oncol.

[116] Mariathasan S, Turley SJ, Nickles D, Castiglioni A, Yuen K, Wang Y (2018). TGFβ attenuates tumour response to PD-L1 blockade by contributing to exclusion of T cells. Nature.

[117] Lin JX, Leonard WJ (2019). Fine-tuning cytokine signals. Annu Rev Immunol.

[118] Li R, Du Y, Chen Z, Xu D, Lin T, Jin S (2020). Macroscopic somatic clonal expansion in morphologically normal human urothelium. Science.

[119] Yoshida K, Gowers KHC, Lee-Six H, Chandrasekharan DP, Coorens T, Maughan EF (2020). Tobacco smoking and somatic mutations in human bronchial epithelium. Nature.

[120] Smola S (2019). Immune deviation and cervical carcinogenesis. Papillomavirus Res (Amsterdam, Netherlands).

[121] Gerdes MJ, Gökmen-Polar Y, Sui Y, Pang AS, LaPlante N, Harris AL (2018). Single-cell heterogeneity in ductal carcinoma in situ of breast. Mod Pathol.

[122] Thompson E, Taube JM, Elwood H, Sharma R, Meeker A, Warzecha HN (2016). The immune microenvironment of breast ductal carcinoma in situ. Mod Pathol.

[123] Srivastava S, Ghosh S, Kagan J, Mazurchuk R, Boja E, Chuaqui R (2018). The making of a precancer atlas: promises, challenges, and opportunities. Trends Cancer.

[124] Rozenblatt-Rosen O, Regev A, Oberdoerffer P, Nawy T, Hupalowska A, Rood JE (2020). The human tumor atlas network: charting tumor transitions across space and time at single-cell resolution. Cell.

[125] Pastushenko I, Blanpain C (2019). EMT transition states during tumor progression and metastasis. Trends Cell Biol.

[126] Puram SV, Tirosh I, Parikh AS, Patel AP, Yizhak K, Gillespie S (2017). Single-cell transcriptomic analysis of primary and metastatic tumor ecosystems in head and neck cancer. Cell.

[127] Derks S, de Klerk LK, Xu X, Fleitas T, Liu KX, Liu Y (2020). Characterizing diversity in the tumor-immune microenvironment of distinct subclasses of gastroesophageal adenocarcinomas. Ann Oncol.

[128] Liu LZ, Zhang Z, Zheng BH, Shi Y, Duan M, Ma LJ (2019). CCL15 recruits suppressive monocytes to facilitate immune escape and disease progression in hepatocellular carcinoma. Hepatology.

[129] Berthel A, Zoernig I, Valous NA, Kahlert C, Klupp F, Ulrich A (2017). Detailed resolution analysis reveals spatial T cell heterogeneity in the invasive margin of colorectal cancer liver metastases associated with improved survival. Oncoimmunology..

[130] Marliot F, Chen X, Kirilovsky A, Sbarrato T, El Sissy C, Batista L, et al. Analytical validation of the Immunoscore and its associated prognostic value in patients with colon cancer. J Immunother Cancer. 2020;8(1). 10.1136/jitc-2019-000272 PMC7253006 32448799

[131] Pagès F, André T, Taieb J, Vernerey D, Henriques J, Borg C (2020). Prognostic and predictive value of the Immunoscore in stage III colon cancer patients treated with oxaliplatin in the prospective IDEA France PRODIGE-GERCOR cohort study. Ann Oncol.

[132] Kather JN, Poleszczuk J, Suarez-Carmona M, Krisam J, Charoentong P, Valous NA (2017). In silico modeling of immunotherapy and stroma-targeting therapies in human colorectal cancer. Cancer Res.

[133] Gallaher JA, Enriquez-Navas PM, Luddy KA, Gatenby RA, Anderson ARA (2018). Spatial heterogeneity and evolutionary dynamics modulate time to recurrence in continuous and adaptive cancer therapies. Cancer Res.

[134] Kaplan RN, Riba RD, Zacharoulis S, Bramley AH, Vincent L, Costa C (2005). VEGFR1-positive haematopoietic bone marrow progenitors initiate the pre-metastatic niche. Nature.

[135] Chin AR, Wang SE (2016). Cancer tills the premetastatic field: mechanistic basis and clinical implications. Clin Cancer Res.

[136] Sceneay J, Smyth MJ, Möller A (2013). The pre-metastatic niche: finding common ground. Cancer Metastasis Rev.

[137] Giles AJ, Reid CM, Evans JD, Murgai M, Vicioso Y, Highfill SL (2016). Activation of hematopoietic stem/progenitor cells promotes immunosuppression within the pre-metastatic niche. Cancer Res.

[138] Clever D, Roychoudhuri R, Constantinides MG, Askenase MH, Sukumar M, Klebanoff CA (2016). Oxygen sensing by T cells establishes an immunologically tolerant metastatic niche. Cell.

[139] Costa-Silva B, Aiello NM, Ocean AJ, Singh S, Zhang H, Thakur BK (2015). Pancreatic cancer exosomes initiate pre-metastatic niche formation in the liver. Nat Cell Biol.

[140] Chen XW, Yu TJ, Zhang J, Li Y, Chen HL, Yang GF (2017). CYP4A in tumor-associated macrophages promotes pre-metastatic niche formation and metastasis. Oncogene.

[141] Liu Y, Gu Y, Han Y, Zhang Q, Jiang Z, Zhang X (2016). Tumor exosomal RNAs promote lung pre-metastatic niche formation by activating alveolar epithelial TLR3 to recruit neutrophils. Cancer Cell.

[142] Peinado H, Zhang H, Matei IR, Costa-Silva B, Hoshino A, Rodrigues G (2017). Pre-metastatic niches: organ-specific homes for metastases. Nat Rev Cancer.

[143] Lin D, Chen X, Lin Z, Lin J, Liu Y, Liu D. Paper-supported co-culture system for dynamic investigating the lung-tropic migration of breast cancer cells. Biomed Mater (Bristol, England). 2020. 10.1088/1748-605X/abc28c 33075760

[144] Roblek M, Calin M, Schlesinger M, Stan D, Zeisig R, Simionescu M (2015). Targeted delivery of CCR2 antagonist to activated pulmonary endothelium prevents metastasis. J Control Release.

[145] García-Caballero M, Van de Velde M, Blacher S, Lambert V, Balsat C, Erpicum C (2017). Modeling pre-metastatic lymphvascular niche in the mouse ear sponge assay. Sci Rep.

[146] Ouzounova M, Lee E, Piranlioglu R, El Andaloussi A, Kolhe R, Demirci MF (2017). Monocytic and granulocytic myeloid derived suppressor cells differentially regulate spatiotemporal tumour plasticity during metastatic cascade. Nat Commun.

[147] Tsai JH, Donaher JL, Murphy DA, Chau S, Yang J (2012). Spatiotemporal regulation of epithelial-mesenchymal transition is essential for squamous cell carcinoma metastasis. Cancer Cell.

[148] Ombrato L, Nolan E, Kurelac I, Mavousian A, Bridgeman VL, Heinze I (2019). Metastatic-niche labelling reveals parenchymal cells with stem features. Nature.

[149] Massagué J, Obenauf AC (2016). Metastatic colonization by circulating tumour cells. Nature.

[150] Zhang AW, McPherson A, Milne K, Kroeger DR, Hamilton PT, Miranda A (2018). Interfaces of malignant and immunologic clonal dynamics in ovarian cancer. Cell.

[151] Angelova M, Mlecnik B, Vasaturo A, Bindea G, Fredriksen T, Lafontaine L (2018). Evolution of metastases in space and time under immune selection. Cell.

[152] Kim TM, Jung SH, An CH, Lee SH, Baek IP, Kim MS (2015). Subclonal genomic architectures of primary and metastatic colorectal cancer based on intratumoral genetic heterogeneity. Clin Cancer Res.

[153] Chang CH, Qiu J, O'Sullivan D, Buck MD, Noguchi T, Curtis JD (2015). Metabolic competition in the tumor microenvironment is a driver of cancer progression. Cell.

[154] Lyssiotis CA, Kimmelman AC (2017). Metabolic interactions in the tumor microenvironment. Trends Cell Biol.

[155] Vaupel P, Kallinowski F, Okunieff P (1989). Blood flow, oxygen and nutrient supply, and metabolic microenvironment of human tumors: a review. Cancer Res.

[156] Vaupel P, Harrison L (2004). Tumor hypoxia: causative factors, compensatory mechanisms, and cellular response. Oncologist.

[157] Viallard C, Larrivée B (2017). Tumor angiogenesis and vascular normalization: alternative therapeutic targets. Angiogenesis.

[158] Dewhirst MW, Ong ET, Klitzman B, Secomb TW, Vinuya RZ, Dodge R (1992). Perivascular oxygen tensions in a transplantable mammary tumor growing in a dorsal flap window chamber. Radiat Res.

[159] Lyng H, Sundfør K, Tropé C, Rofstad EK (1996). Oxygen tension and vascular density in human cervix carcinoma. Br J Cancer.

[160] Yeom CJ, Goto Y, Zhu Y, Hiraoka M, Harada H (2012). Microenvironments and cellular characteristics in the micro tumor cords of malignant solid tumors. Int J Mol Sci.

[161] Helmlinger G, Yuan F, Dellian M, Jain RK (1997). Interstitial pH and pO2 gradients in solid tumors in vivo: high-resolution measurements reveal a lack of correlation. Nat Med.

[162] Vaupel P, Fortmeyer HP, Runkel S, Kallinowski F (1987). Blood flow, oxygen consumption, and tissue oxygenation of human breast cancer xenografts in nude rats. Cancer Res.

[163] Milotti E, Fredrich T, Chignola R, Rieger H (2020). Oxygen in the tumor microenvironment: mathematical and numerical modeling. Adv Exp Med Biol.

[164] Cao X, Allu SR, Jiang S, Gunn Bs JR, Yao Ph DC, Xin Ph DJ (2021). High-resolution pO2 imaging improves quantification of the hypoxic fraction in tumors during radiation therapy. Int J Radiat Oncol Biol Phys.

[165] Kasinskas RW, Venkatasubramanian R, Forbes NS (2014). Rapid uptake of glucose and lactate, and not hypoxia, induces apoptosis in three-dimensional tumor tissue culture. Integr Biol Quant Biosci Nano Macro.

[166] Riera-Domingo C, Audigé A, Granja S, Cheng WC, Ho PC, Baltazar F (2020). Immunity, hypoxia, and metabolism-the ménage à trois of cancer: implications for immunotherapy. Physiol Rev.

[167] Laoui D, Van Overmeire E, Di Conza G, Aldeni C, Keirsse J, Morias Y (2014). Tumor hypoxia does not drive differentiation of tumor-associated macrophages but rather fine-tunes the M2-like macrophage population. Cancer Res.

[168] Takeda N, O'Dea EL, Doedens A, Kim JW, Weidemann A, Stockmann C (2010). Differential activation and antagonistic function of HIF-{alpha} isoforms in macrophages are essential for NO homeostasis. Genes Dev.

[169] Henze AT, Mazzone M (2016). The impact of hypoxia on tumor-associated macrophages. J Clin Invest.

[170] Franklin RA, Liao W, Sarkar A, Kim MV, Bivona MR, Liu K (2014). The cellular and molecular origin of tumor-associated macrophages. Science.

[171] Taylor CT, Colgan SP (2017). Regulation of immunity and inflammation by hypoxia in immunological niches. Nat Rev Immunol.

[172] Dehne N, Mora J, Namgaladze D, Weigert A, Brüne B (2017). Cancer cell and macrophage cross-talk in the tumor microenvironment. Curr Opin Pharmacol.

[173] Kumar V, Gabrilovich DI (2014). Hypoxia-inducible factors in regulation of immune responses in tumour microenvironment. Immunology.

[174] Kim BJ, Forbes NS (2008). Single-cell analysis demonstrates how nutrient deprivation creates apoptotic and quiescent cell populations in tumor cylindroids. Biotechnol Bioeng.

[175] Wyss MT, Hofer S, Hefti M, Bärtschi E, Uhlmann C, Treyer V (2007). Spatial heterogeneity of low-grade gliomas at the capillary level: a PET study on tumor blood flow and amino acid uptake. J Nucl Med.

[176] Leslie TK, James AD, Zaccagna F, Grist JT, Deen S, Kennerley A (2019). Sodium homeostasis in the tumour microenvironment. Biochim Biophys Acta.

[177] Tan JWY, Folz J, Kopelman R, Wang X (2020). In vivo photoacoustic potassium imaging of the tumor microenvironment. Biomed Opt Express.

[178] Bian Y, Li W, Kremer DM, Sajjakulnukit P, Li S, Crespo J (2020). Cancer SLC43A2 alters T cell methionine metabolism and histone methylation. Nature.

[179] Cascone T, McKenzie JA, Mbofung RM, Punt S, Wang Z, Xu C (2018). Increased tumor glycolysis characterizes immune resistance to adoptive T cell therapy. Cell Metab.

[180] Galon J, Fridman WH, Pages F (2007). The adaptive immunologic microenvironment in colorectal cancer: a novel perspective. Cancer Res.

[181] Angell H, Galon J (2013). From the immune contexture to the Immunoscore: the role of prognostic and predictive immune markers in cancer. Curr Opin Immunol.

[182] Pagès F, Mlecnik B, Marliot F, Bindea G, Ou FS, Bifulco C (2018). International validation of the consensus Immunoscore for the classification of colon cancer: a prognostic and accuracy study. Lancet.

[183] Van den Eynde M, Mlecnik B, Bindea G, Fredriksen T, Church SE, Lafontaine L (2018). The link between the multiverse of immune microenvironments in metastases and the survival of colorectal cancer patients. Cancer Cell.

[184] Fortis SP, Sofopoulos M, Sotiriadou NN, Haritos C, Vaxevanis CK, Anastasopoulou EA (2017). Differential intratumoral distributions of CD8 and CD163 immune cells as prognostic biomarkers in breast cancer. J Immunother Cancer.

[185] Pagès F, Kirilovsky A, Mlecnik B, Asslaber M, Tosolini M, Bindea G (2009). In situ cytotoxic and memory T cells predict outcome in patients with early-stage colorectal cancer. J Clin Oncol.

[186] Barua S, Fang P, Sharma A, Fujimoto J, Wistuba I, Rao AUK (2018). Spatial interaction of tumor cells and regulatory T cells correlates with survival in non-small cell lung cancer. Lung Cancer (Amsterdam, Netherlands).

[187] Mahmoud SM, Paish EC, Powe DG, Macmillan RD, Grainge MJ, Lee AH (2011). Tumor-infiltrating CD8+ lymphocytes predict clinical outcome in breast cancer. J Clin Oncol.

[188] Mezheyeuski A, Bergsland CH, Backman M, Djureinovic D, Sjöblom T, Bruun J (2018). Multispectral imaging for quantitative and compartment-specific immune infiltrates reveals distinct immune profiles that classify lung cancer patients. J Pathol.

[189] Enfield KSS, Martin SD, Marshall EA, Kung SHY, Gallagher P, Milne K (2019). Hyperspectral cell sociology reveals spatial tumor-immune cell interactions associated with lung cancer recurrence. J Immunother Cancer.

[190] Dieu-Nosjean MC, Antoine M, Danel C, Heudes D, Wislez M, Poulot V (2008). Long-term survival for patients with non-small-cell lung cancer with intratumoral lymphoid structures. J Clin Oncol.

[191] Dieu-Nosjean MC, Goc J, Giraldo NA, Sautès-Fridman C, Fridman WH (2014). Tertiary lymphoid structures in cancer and beyond. Trends Immunol.

[192] Sofopoulos M, Fortis SP, Vaxevanis CK, Sotiriadou NN, Arnogiannaki N, Ardavanis A (2019). The prognostic significance of peritumoral tertiary lymphoid structures in breast cancer. Cancer Immunol Immunother.

[193] García-Hernández ML, Uribe-Uribe NO, Espinosa-González R, Kast WM, Khader SA, Rangel-Moreno J (2017). A unique cellular and molecular microenvironment is present in tertiary lymphoid organs of patients with spontaneous prostate cancer regression. Front Immunol.

[194] Doherty ML (1987). Laminitis in beef bulls. Vet Rec.

[195] Kagen LJ, Gurevich R (1967). Precipitin reactions of anti-human myoglobin serum with several human and animal muscle extracts. Immunology.

[196] Karaca Z, Tanriverdi F, Unluhizarci K, Ozturk F, Gokahmetoglu S, Elbuken G (2011). VEGFR1 expression is related to lymph node metastasis and serum VEGF may be a marker of progression in the follow-up of patients with differentiated thyroid carcinoma. Eur J Endocrinol.

[197] Xu WW, Li B, Lam AK, Tsao SW, Law SY, Chan KW (2015). Targeting VEGFR1- and VEGFR2-expressing non-tumor cells is essential for esophageal cancer therapy. Oncotarget.

[198] Liu X, Bao X, Hu M, Chang H, Jiao M, Cheng J, et al. Inhibition of PCSK9 potentiates immune checkpoint therapy for cancer. Nature. 2020. 10.1038/s41586-020-2911-7 PMC7770056 33177715

[199] Imtiyaz HZ, Williams EP, Hickey MM, Patel SA, Durham AC, Yuan LJ (2010). Hypoxia-inducible factor 2alpha regulates macrophage function in mouse models of acute and tumor inflammation. J Clin Invest.

[200] Chen J, Luo H, Liu Y, Zhang W, Li H, Luo T (2017). Oxygen-self-produced nanoplatform for relieving hypoxia and breaking resistance to sonodynamic treatment of pancreatic cancer. ACS Nano.

[201] Liu H, Jiang W, Wang Q, Hang L, Wang Y, Wang Y (2019). ROS-sensitive biomimetic nanocarriers modulate tumor hypoxia for synergistic photodynamic chemotherapy. Biomater Sci.

[202] Fukumura D, Kloepper J, Amoozgar Z, Duda DG, Jain RK (2018). Enhancing cancer immunotherapy using antiangiogenics: opportunities and challenges. Nat Rev Clin Oncol.

[203] Alonso-Nocelo M, Raimondo TM, Vining KH, López-López R, de la Fuente M, Mooney DJ (2018). Matrix stiffness and tumor-associated macrophages modulate epithelial to mesenchymal transition of human adenocarcinoma cells. Biofabrication.

[204] Najafi M, Farhood B, Mortezaee K (2019). Extracellular matrix (ECM) stiffness and degradation as cancer drivers. J Cell Biochem.

[205] Zhong W, Weiss HL, Jayswal RD, Hensley PJ, Downes LM, St Clair DK (2018). Extracellular redox state shift: a novel approach to target prostate cancer invasion. Free Radical Biol Med.

[206] Marusyk A, Tabassum DP, Janiszewska M, Place AE, Trinh A, Rozhok AI (2016). Spatial proximity to fibroblasts impacts molecular features and therapeutic sensitivity of breast cancer cells influencing clinical outcomes. Can Res.

[207] Kook YM, Jeong Y, Lee K, Koh WG (2017). Design of biomimetic cellular scaffolds for co-culture system and their application. J Tissue Eng.

[208] Tuveson D, Clevers H (2019). Cancer modeling meets human organoid technology. Science.

[209] Neal JT, Li X, Zhu J, Giangarra V, Grzeskowiak CL, Ju J (2018). Organoid modeling of the tumor immune microenvironment. Cell.

[210] Yuki K, Cheng N, Nakano M, Kuo CJ (2020). Organoid models of tumor immunology. Trends Immunol.

[211] Bedognetti D, Ceccarelli M, Galluzzi L, Lu R, Palucka K, Samayoa J (2019). Toward a comprehensive view of cancer immune responsiveness: a synopsis from the SITC workshop. J Immunother Cancer.

[212] Lau D, Garçon F, Chandra A, Lechermann L, Aloj L, Chilvers E (2020). Intravital imaging of adoptive t-cell morphology, mobility and trafficking following immune checkpoint inhibition in a mouse melanoma model. Front Immunol.

